# Biotechnology-Enabled Food Reformulation for Prevention of Metabolic Syndrome: Nutrient and Dietary Pattern Strategies

**DOI:** 10.3390/foods15203707

**Published:** 2026-10-18

**Authors:** Dilyar Tuigunov, Yerzhan Omarov, Akkumis Salkhanova, Galiya Smagul, Malika Abdulzhanova, Oksana Dolmatova

**Affiliations:** 1 Laboratory of Food Biotechnology and Specialized Food Products, Kazakh Academy of Nutrition, Almaty 050008, Kazakhstan; dilyar117@gmail.com (D.T.); yerzhan_omarov@mail.ru (Y.O.); asalkhanova@academypm.org (A.S.); 2 Department of Food Safety and Quality, Faculty of Food Technology, Almaty Technological University, Almaty 050012, Kazakhstan; s.galiya_22@mail.ru; 3 Department of Biotechnology, Faculty of Biology and Biotechnology, Al-Farabi Kazakh National University, Almaty 050040, Kazakhstan; malika.abdulzhanova@kaznu.edu.kz

**Keywords:** metabolic syndrome, food reformulation, food biotechnology, bioactive compounds, dietary patterns

## Abstract

Metabolic syndrome is a cluster of cardiometabolic risk factors in which modifiable dietary factors play a substantial role. This structured narrative review summarizes evidence on biotechnological food reformulation, defined as the modification of the composition or functional properties of foods already present in the diet using enzymes, microorganisms, fermentation, and bioconversion. Approaches to reducing free sugar and sodium content, modifying lipid and protein components, and increasing micronutrient content and bioavailability are reviewed. The data were analyzed with a distinction between experimental systems (in vitro, animal, and human intervention and observational studies) and study populations (healthy individuals, individuals with risk factors, and patients with diagnosed metabolic diseases). Mechanistic and animal data support the biological plausibility of the effects of several reformulated products on individual cardiometabolic markers. However, human intervention studies are few and mostly short-term. They rely on surrogate markers and are often conducted in patients with established disorders; therefore, they do not allow conclusions to be drawn about preventive efficacy. Consumer acceptance, safety, regulatory status, and industrial scalability are discussed on the basis of limited and heterogeneous data and require separate assessment for each technology. Biotechnological reformulation may be considered a promising approach to improving the nutrient profile of mass-market foods. However, its impact on the risk of metabolic syndrome has not yet been established and should be evaluated in long-term randomized trials in individuals without diagnosed metabolic diseases.

## 1. Introduction

Metabolic syndrome (MetS) is a cluster of modifiable risk factors associated with the development of cardiovascular disease, type 2 diabetes mellitus, and other adverse health outcomes, which makes it relevant to both clinical practice and public health [1,2]. According to the World Health Organization (WHO) criteria, this pathological condition, also known as “syndrome X” or “insulin resistance syndrome”, is characterized by a set of interrelated disorders, the key ones being abdominal obesity, insulin resistance, arterial hypertension, and dyslipidemia [3].

The prevalence of overweight and obesity, which are key drivers and components of MetS, has reached the scale of a global non-communicable disease epidemic [4]. According to meta-analyses, the prevalence of MetS varies considerably, ranging from 10% to 84% depending on the ethnic, racial, age, and sex characteristics of the populations studied [5]. MetS, with a mean prevalence of 31%, is associated with a twofold increase in the risk of coronary heart disease and cerebrovascular disease, as well as a 1.5-fold increase in the risk of all-cause mortality [6].

The etiology of MetS is largely determined by modifiable lifestyle factors, among which diet plays a key role [7]. In particular, excessive consumption of energy-dense foods rich in saturated fatty acids and simple carbohydrates, as well as adherence to a Western dietary pattern, is associated with a 30% increase in the risk of obesity [8]. Conversely, adequate intake of vitamins, minerals, whole grains, dairy products, fruits, and vegetables as part of a balanced diet is associated with a more favorable metabolic profile and a lower risk of MetS [9,10].

Recent studies have identified consistent longitudinal associations between characteristics of the retail food environment and poorer diet quality, rising obesity rates, and the risk of individual components of MetS [11]. However, transforming the food environment, particularly toward greater availability and appeal of healthy foods, faces considerable technological and economic challenges. Conventional strategies focused primarily on modifying individual behavior have substantial limitations, including poor long-term adherence to recommendations and the strong influence of the food environment [12].

Despite the growing body of evidence on the influence of dietary factors on MetS risk, practice-oriented strategies for the targeted transformation of the food environment to improve population health are currently lacking. The resulting scientific and technological gap lies in the insufficient understanding of the mechanisms of specific tools for developing next-generation food products for the prevention of key components of MetS [13].

Overcoming these barriers comprehensively, at the level of both individual behavioral choices and systemic constraints, requires the development and implementation of innovative strategies. In this context, biotechnological approaches that provide tools for the targeted modification of food products are of particular importance. One of the key directions is the biotechnological reformulation of mass-market foods aimed at improving their nutrient profile without compromising their sensory and consumer properties [14]. Implementing this approach may contribute to a healthier food environment and could potentially serve as one component of the primary prevention of MetS; however, the magnitude of these effects depends on product composition, the food matrix, intake levels, and confirmation in clinical and population-based studies.

Biotechnology is regarded as one of the strategic drivers of sustainable improvement in quality of life, contributing to food security, a reduced burden of chronic non-communicable diseases, and the environmentally responsible application of molecular and engineering technologies oriented toward the long-term needs of society [15]. Given these capabilities, it appears appropriate to focus on the applied biotechnological tools aimed at modifying the composition and functional properties of foods that are widely consumed by the population. Current biotechnological approaches aim to reformulate foods by reducing their energy content, optimizing their micronutrient profile, and fortifying them with bioactive components, thereby preserving their sensory appeal to consumers while enhancing their nutritional value [16].

Previously published reviews have characterized functional foods for MetS without differentiating the technologies used to produce them, evaluated barriers to reformulation in the food industry without linking them to metabolic outcomes, or summarized data on the bioproduction of food ingredients mainly from a technological perspective [17,18]. However, it remains insufficiently understood which biotechnological approaches to food reformulation have been supported by human studies in the very food form in which the corresponding products are intended to be consumed.

The novelty of this review lies in comparing biotechnological approaches to food reformulation across major nutrient targets and components of MetS, taking into account the level of evidence and distinguishing between studies conducted on actual food products, on isolated components, and in preclinical models. The aim of this review is to evaluate the strength of evidence supporting biotechnological reformulation as a tool for MetS prevention and to determine which technologies are applicable to modifying key nutritional characteristics of foods, through which mechanisms they may affect the components of MetS, to what extent their effects have been studied in humans using actual food products, and which aspects remain insufficiently explored.

In addition to biotechnological processes per se, this review considers bioactive food components—including polyunsaturated fatty acids (PUFAs), vitamins, polyphenols, and probiotics—and dietary patterns. Each of these categories serves a specific analytical function within the reformulation framework under consideration. PUFAs, vitamins, and polyphenols are considered potential target ingredients for biotechnological reformulation, since their production (e.g., microbial synthesis of PUFAs and carotenoids) or structural modification (e.g., enzymatic biotransformation of polyphenol glycosides into aglycones) can be achieved using biotechnological approaches; fortification with these components is not in itself a biotechnological process. Data on their physiological effects are used to justify the selection of potential nutrient targets and ingredients but are not regarded as independent evidence of the efficacy of reformulated foods.

Probiotics, being live microorganisms, are considered a separate category of biotechnological components potentially applicable in the development of reformulated foods. Dietary patterns are analyzed mainly as a context for identifying priority nutrient targets and product categories for reformulation. Notably, within the scope of this review, oleogelation and encapsulation are not classified as biotechnological approaches; rather, they are considered solely as auxiliary (enabling) technological tools for the stabilization and protection of ingredients of biotechnological origin within the food matrix.

## 2. Search and Review Methodology

### 2.1. Review Design and Scope

This structured narrative review focuses on biotechnological approaches to food reformulation for the prevention of MetS. It is based on a structured literature search and a defined study selection procedure, which ensures transparency in building the evidence base while preserving the interpretive and integrative nature of narrative analysis. No quantitative data synthesis was performed.

The review addresses four interrelated questions: (i) which biotechnological strategies are applicable to the modification of key nutrients, and through which mechanisms they may affect the components of MetS; (ii) to what extent their effects have been confirmed in human studies using reformulated foods; (iii) how the level of evidence differs between in vitro, animal, and human studies, as well as between preventive and therapeutic interventions; and (iv) which factors limit the practical implementation of reformulated products.

In this review, biotechnological reformulation is defined as the modification of the composition, structure, or functional properties of a food product using biological systems or their components, including enzymes, microorganisms, fermentation and bioconversion processes, and the biofortification of food crops.

Physicochemical structuring and delivery technologies, including oleogelation, physical lipid structuring, and encapsulation, are not biotechnologies per se. In this review, they were treated as enabling technologies and were included in only two cases: (i) when they were applied to stabilize and protect within the food matrix ingredients of biotechnological origin (probiotic microorganisms, postbiotics, bioactive peptides, enzymatically produced structured lipids, microbial oils, and fermentation products); or (ii) when they were combined with a biotechnological process within a single reformulation strategy, for example, in the development of oleogels based on enzymatically modified lipids or microbially derived biopolymers.

Thus, reformulation is regarded as a technological chain encompassing the biotechnological production of a functional ingredient, its stabilization within the food matrix, and the retention of its functional properties in the final product.

### 2.2. Literature Search Strategy

Literature searches were conducted in Scopus, Web of Science Core Collection, and PubMed. Supplementary searches were performed in ScienceDirect and Google Scholar. The search queries were last updated on 15 May 2026. The main search period covered publications from 1 January 2000 to the date of the last update. Earlier seminal studies were included where they were necessary to describe mechanisms of action or the technological development of the approaches under consideration, or to interpret current evidence.

The search strategy comprised three complementary domains:

(i) Biotechnological approaches to food reformulation in relation to MetS and its components.
(ii) The effects of bioactive components used, or potentially applicable, in reformulated products on metabolic, inflammatory, and microbiota-related markers of MetS.
(iii) The integration of reformulated products into dietary patterns, as well as issues of safety, regulatory status, and consumer acceptance.

The main groups of Boolean search strings were as follows:

- (“metabolic syndrome” OR “insulin resistance” OR obesity OR dyslipidemia OR “arterial hypertension” OR “cardiometabolic health” OR “type 2 diabetes”) AND (“food reformulation” OR “biotechnology-enabled food reformulation” OR “food biotechnology” OR “sugar reduction” OR “rare sugar*” OR allulose OR tagatose OR “functional oligosaccharide*” OR “resistant starch” OR “sodium reduction” OR “salt substitute*” OR “salt reduction” OR “enzymatic interesterification” OR “structured lipid*” OR oleogel* OR “protein hydrolysate*” OR “enzymatic protein hydrolysis” OR “bioactive peptide*” OR biofortification OR encapsulation);
- (probiotic* OR synbiotic* OR postbiotic* OR “dietary fiber” OR prebiotic* OR “omega-3” OR “polyunsaturated fatty acid*” OR vitamin* OR mineral* OR polyphenol* OR carotenoid*) AND (“metabolic syndrome” OR “insulin resistance” OR “glycemic control” OR “lipid profile” OR “blood pressure” OR inflammation OR “gut microbiota”) AND (food* OR “food matrix” OR “fortified food*” OR “fermented food*” OR “functional food*”);
- (“dietary pattern*” OR “Mediterranean diet” OR “DASH diet” OR “Nordic diet”) AND (“food reformulation” OR “reformulated food*” OR “functional food*”) AND (“metabolic syndrome” OR “cardiometabolic risk”).

The search syntax was adapted to each database. In Scopus, searches were performed in the TITLE-ABS-KEY fields; in Web of Science Core Collection, in the Topic field; and in PubMed, in the Title/Abstract fields. In ScienceDirect and Google Scholar, equivalent combinations of keywords and Boolean operators were applied using the standard search interfaces. Where necessary, search strategies were refined by combining the names of specific technologies with the type of food matrix or the metabolic outcome of interest.

### 2.3. Eligibility Criteria

Studies were included in this review if they met the following criteria: (i) original experimental, clinical, and observational studies, as well as systematic reviews and meta-analyses, addressing biotechnological approaches to food reformulation or bioactive components potentially applicable in reformulated food matrices; (ii) studies aimed at modifying the nutrient composition, food matrix structure, functional properties, or bioavailability of food components; (iii) studies assessing the effects of reformulated products, individual bioactive components, or dietary patterns on MetS risk factors, including body weight and body composition, waist circumference, indices of glycemic control and insulin resistance, lipid profile, blood pressure, markers of inflammation and oxidative stress, and the composition and metabolic activity of the gut microbiota; (iv) studies on the application of biotechnological processes (enzymatic modification, microbial fermentation, bioconversion, and biofortification), as well as studies in which physicochemical structuring and delivery technologies (oleogelation and encapsulation) were applied to ingredients of biotechnological origin or combined with biotechnological processes; and (v) studies reporting data on the safety, sensory properties, storage stability, regulatory status, or technological scalability of reformulated food products. The review primarily included full-text, peer-reviewed publications in English indexed in the selected international bibliographic databases. Preference was given to studies that clearly described the composition of both the original and the reformulated product, the biotechnological approach applied, the study design, the characteristics of the model or participants, the dose and duration of the intervention, and quantitative metabolic outcomes. Studies reporting negative or null results were included on an equal basis with those reporting beneficial effects, provided that they met the other criteria.

Studies were excluded if they met at least one of the following criteria: (i) publications unrelated to biotechnological food reformulation, food bioactive components, or the prevention of MetS; (ii) studies focusing exclusively on physicochemical structuring, emulsification, or encapsulation technologies (including oleogelation and microencapsulation) without a biotechnological component, i.e., where the technology was neither applied to ingredients of biotechnological origin nor combined with a biotechnological process; (iii) studies focusing exclusively on pharmacological agents or medical interventions unrelated to foods or nutrients; (iv) studies focusing exclusively on conventional processing, storage, or sensory properties of foods without assessment of their nutrient composition, functional properties, or metabolic relevance; (v) publications with insufficient description of the methodology, product composition, intervention parameters, or outcomes required for interpretation; (vi) conference abstracts, patents, dissertations, editorials, commentaries, and other non-peer-reviewed sources; and (vii) duplicate publications or studies based on identical datasets that provided no additional information relevant to the aims of this review.

### 2.4. Study Selection Process

The search results were merged into a single bibliographic library. Duplicate records were identified automatically and additionally verified manually. Study selection was performed in three stages: (i) removal of duplicates and records clearly outside the scope of the review; (ii) title and abstract screening; and (iii) full-text assessment of potentially relevant publications against the inclusion and exclusion criteria.

A publication was considered potentially relevant if it addressed one or more of the following areas:

- Biotechnological reduction in free sugars, sodium, saturated fatty acids, and trans fatty acids in food products.
- Biotechnological modification of the protein fraction and production of bioactive peptides.
- Enhancement of the bioavailability and stability of micronutrients and phytochemicals in food matrices.
- Effects of probiotics, synbiotics, postbiotics, dietary fiber, polyunsaturated fatty acids, vitamins, minerals, polyphenols, and carotenoids on the components of MetS.
- Integration of reformulated products into dietary patterns.
- Safety, regulatory status, consumer acceptance, and technological feasibility of reformulated products.

At the full-text assessment stage, studies were excluded if they lacked a link to food reformulation or to the components of MetS, applied physicochemical technologies without a biotechnological component, did not report relevant metabolic outcomes, or provided an insufficient description of the product composition, intervention parameters, or results. Additional publications were identified by screening the reference lists of the included studies. Following the selection process, a total of 259 sources were included in the final review, comprising original experimental, clinical, and observational studies and review articles, which were subsequently subjected to qualitative synthesis.

Studies selected for inclusion in the summary tables were those that directly illustrated each reformulation strategy or each category of bioactive components under consideration. Priority was given to the highest level of evidence available for each strategy. Where human studies using actual food products were available, they were included first; in their absence, preclinical studies were included, with the type of experimental system specified.

### 2.5. Data Extraction and Evidence Synthesis

The following data were extracted from the included original studies: the type of food product or matrix, the biotechnological approach applied, the reformulated component, the intervention category, the experimental model or participant characteristics, the dose and duration of the intervention, the metabolic outcomes assessed, the main findings, and the study limitations.

Studies were classified according to the type of experimental system: (i) cell-free in vitro experiments, including chemical and enzymatic assays and simulated gastrointestinal digestion models; (ii) cell-based in vitro experiments; (iii) animal studies; (iv) human intervention studies; and (v) human observational studies (cohort, case–control, and cross-sectional). For animal studies, additional data were extracted on the animal species and strain, the method used to induce metabolic disorders (high-fat or high-carbohydrate diet, genetic models), and the duration of the experiment. For human intervention studies, data were extracted on the study design (randomized controlled, crossover, open-label), the number and characteristics of participants, baseline metabolic status, and the dose and duration of the intervention. Data from preclinical studies were interpreted as mechanistic or hypothesis-generating and were not directly extrapolated to clinical efficacy in humans.

Interventions were classified into three categories, which were not considered equivalent: (i) reformulated food products with biotechnologically modified composition or structure, which constituted the primary focus of the analysis; (ii) isolated bioactive compounds and dietary supplements, data on which were used to support their potential use as ingredients in reformulated food matrices; and (iii) conventional foods and dietary patterns, considered as the context for the implementation of reformulated products. In addition, a distinction was made between preventive effects observed in healthy individuals and individuals with MetS risk factors, and therapeutic effects observed in patients with diagnosed MetS, type 2 diabetes mellitus, or other metabolic diseases. Data obtained from patients with diagnosed MetS, type 2 diabetes mellitus, or other metabolic disorders were interpreted as pertaining to a therapeutic context and were not used as direct evidence of preventive efficacy; for each human study described, the baseline metabolic status of the participants is specified.

For each reformulation strategy, the availability and nature of data on realistically achievable intake levels from food products, safety, sensory properties, storage stability, and cost were additionally assessed. Where such data were unavailable, this was explicitly stated.

Owing to the substantial heterogeneity of food matrices, biotechnological approaches, experimental models, study designs, doses, intervention durations, and outcomes assessed, no statistical pooling of results or meta-analysis was performed. Evidence was synthesized qualitatively, with an emphasis on identifying common patterns, matrix-dependent effects, conflicting and null results, and gaps in the evidence base. Particular attention was paid to strategies for which human studies using actual food products are lacking or scarce.

## 3. Multilevel Mechanisms of Food Reformulation: Nutrient Sensing, Gut Microbiota, and Behavioral Regulation

**Figure 1:**
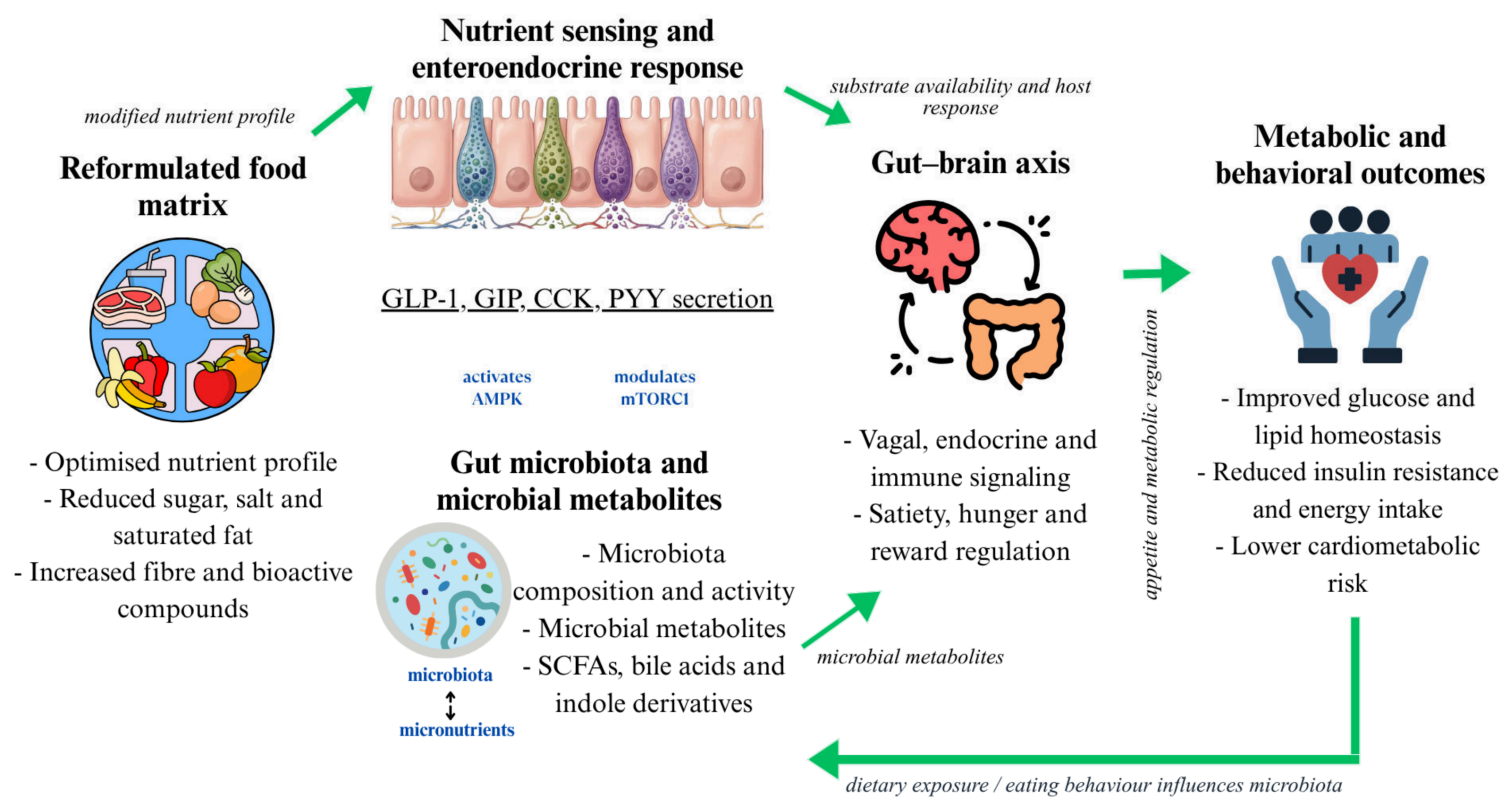
Conceptual framework of potential mechanisms linking biotechnological food reformulation with cardiometabolic outcomes. The scheme integrates nutrient sensing, gut microbiota and microbial metabolites, and gut–brain signaling as potential pathways through which reformulation may influence metabolic and behavioral responses. Solid, dashed, and dotted arrows denote relationships supported by human and/or experimental evidence, predominantly preclinical evidence, and hypothetical or insufficiently validated mechanisms, respectively. The depicted metabolic outcomes represent potential associations rather than established causal effects or evidence of MetS prevention. The framework is intended to distinguish mechanistic plausibility from demonstrated effects of reformulated foods and highlights the need for validation in long-term human studies.

Public health-oriented food reformulation requires the complete replacement of products on the market rather than an expansion of the product range [19]. Predicting and managing the consequences of a systemic shift in dietary patterns through food reformulation requires a systems-level analysis of how modified nutrient compositions within a specific food matrix interact with nutrient-sensing systems and modulate the composition of the gut microbiota, thereby driving changes in eating behavior and metabolic health (Figure 1). It should be emphasized that the mechanisms discussed in this section have been established mainly in cell-based in vitro experiments and animal studies and summarized in reviews; findings from human studies are reported separately.

Extraoral sweet taste receptors of the T1R family play a key role in this process [20,21]. These receptors are expressed in various tissues; in intestinal enteroendocrine cells, they are activated by glucose and stimulate the secretion of the incretins glucagon-like peptide-1 (GLP-1) and glucose-dependent insulinotropic polypeptide (GIP), thereby contributing to the regulation of glycemic and lipid homeostasis [22,23,24,25]. T1R receptors are linked to intracellular energy sensors, such as mechanistic target of rapamycin complex 1 (mTORC1), AMP-activated protein kinase (AMPK), and general control nonderepressible 2 (GCN2) kinase, which may account for differences in the metabolic response to foods with a modified nutrient composition. mTORC1 integrates nutrient signals to regulate cellular metabolism and systemic homeostasis, and its dysregulation is considered a pathogenetic basis of MetS and type 2 diabetes mellitus [26,27,28,29]. AMPK, a central energy sensor, is activated by an increased AMP/ATP ratio [30] and modulates tissue-specific pathways in different directions: it stimulates fatty acid oxidation and suppresses lipogenesis in the liver, inhibits lipolysis in adipocytes, and regulates insulin synthesis in pancreatic β-cells [31,32,33,34]. In addition to its homeostatic role under nutrient deprivation, GCN2 is potentially involved in the pathogenesis of MetS through the regulation of inflammation, insulin signaling, and lipid metabolism, although the molecular basis of this involvement remains poorly understood [35,36]. Experimental data suggest that these sensors may function as an interconnected network: activation of T1R receptors can modulate the mTORC1, AMPK, and GCN2 signaling pathways, pointing to crosstalk between membrane receptor signaling and intracellular nutrient sensing; however, these interrelationships have not been confirmed in humans.

These processes are not limited to the cellular and tissue levels of regulation. Within a complex food matrix, a substantial proportion of nutrients and their metabolites undergo further biotransformation in the gut, where the gastrointestinal microbial community plays a key role [37]. The gut microbiota modifies nutrient bioavailability and produces a wide range of signaling metabolites, thereby indirectly influencing nutrient-sensing mechanisms [38]. Findings from cell-based in vitro experiments and animal studies, summarized in reviews, demonstrate that gut microorganisms and their metabolites, including short-chain fatty acids (SCFAs), secondary bile acids, and indole and phenolic compounds, can regulate host metabolic and immune responses by modulating intracellular signaling pathways, including AMPK- and mTORC1-dependent mechanisms [39,40]. In addition to producing signaling molecules, commensal microorganisms serve as an endogenous source of essential micronutrients, modulating the host’s vitamin and trace element status [41]. This interaction highlights the bidirectional nature of the microbiota–micronutrient axis, in which the composition of the microbial community determines nutrient availability, while micronutrient status, in turn, affects the taxonomic and functional diversity of the microbiota [42]. Furthermore, the gut microbiota has been reported to participate in the regulation of host metabolism, whereas metabolic disorders, including obesity, are associated with gut dysbiosis [43,44]. This microbiota-mediated regulation of micronutrient homeostasis and the associated dysbiotic shifts in key metabolic disorders open up prospects for food reformulation aimed at MetS prevention. Alterations in the micronutrient composition of foods may affect gut microbiota composition and the bioavailability of essential elements; however, the association of such alterations with metabolic disorders related to insulin resistance and obesity has been demonstrated predominantly in experimental studies [45,46].

The impact of reformulation extends beyond metabolic and microbial interactions, as these mechanisms ultimately mediate the regulation of food intake at the whole-body level [47]. Nutrient-sensing signals, enteroendocrine responses, and microbial metabolites contribute to the perception of hunger and satiety, food motivation, and persistent patterns of eating behavior [48]. Within the framework of the gut–brain axis, signals generated in response to a reformulated product are transmitted from the intestinal epithelium and enteroendocrine cells to regulatory centers in the brain [49]. Changes in the fatty acid profile, protein composition, and food matrix structure can modify the rate of gastric emptying and the secretion of the gut hormones cholecystokinin, GLP-1, and peptide YY (PYY), as demonstrated, for example, in a human intervention study comparing fat emulsions with different digestion rates [50]. Together, these factors determine not only the metabolic response to a meal but also the degree of subjective satiety, the time interval until the next meal, the motivational value of the product, and the likelihood of excess energy intake [51].

Thus, food reformulation constitutes a modification of product composition whose effects may potentially manifest at multiple levels, ranging from nutrient sensing and enteroendocrine signaling to microbial–metabolic interactions and eating behavior [52]. It is the integration of data on nutrient sensing, the gut microbiota, and behavioral regulation that provides the theoretical and experimental basis for developing functional foods with the potential to influence eating behavior and metabolic markers. However, the persistence of such effects and their clinical relevance require confirmation in long-term human studies.

## 4. Biotechnological Approaches to the Reformulation of Key Nutrients for Prevention of Metabolic Syndrome

### 4.1. Enzymatic Strategies for Reducing the Content of Free Sugars

Excessive intake of free sugars, particularly sucrose, has been identified in reviews as a key dietary risk factor contributing to insulin resistance, visceral obesity, and dyslipidemia, which together form the pathogenetic basis of MetS [53]. However, a systematic review of intervention studies in healthy adults found that the adverse metabolic effects of sucrose occur predominantly at high levels of intake [54]. A review of epidemiological (observational) and intervention studies reported an association between high dietary intake of added sugars and an increased risk of non-alcoholic fatty liver disease (NAFLD), cardiovascular disease, and type 2 diabetes mellitus, often independent of weight gain and total energy intake [55]. The key pathophysiological effects of free sugars on the components of MetS are summarized in Table 1.

In line with global recommendations to limit free sugar intake to less than 10% of total energy intake, as well as national targets for reducing sugar consumption, there is a need for effective technological solutions that enable a substantial reduction in the sugar content of mass-market foods without compromising their sensory and consumer properties [56,57]. The direct replacement of sucrose with high-intensity sweeteners, such as aspartame, sucralose, and steviol glycosides, is often associated with technological and physiological limitations. These include changes in the sensory profile, such as off-flavors and a temporal sweetness profile that differs from that of sucrose, and, in some cases, adverse effects on the glycemic response and the gut microbiota [58]. However, the effects of low-calorie sweeteners on the gut microbiota have been demonstrated mainly in animal studies, whereas evidence from human studies remains limited and inconsistent [59].

**Table 1:** The effect of free sugars on the pathogenetic components of MetS.

Component of MetS	Pathophysiological Effects of Free Sugars	Clinico-Metabolic Manifestations	References
Insulin resistance	Fructose-induced insulin resistance associated with impaired insulin signaling in the liver and peripheral tissues	Hyperinsulinemia, elevated fasting glucose and insulin levels, reduced insulin sensitivity indices	[60]
Visceral obesity	Fructose-induced inflammation in subcutaneous adipose tissue, liver, and brain, accompanied by increased intracellular cortisol regeneration	Visceral adiposity, hepatic steatosis, TOFI phenotype (“thin outside, fat inside”)	[61]
Dyslipidemia	Free sugar intake is associated with lipid profile disturbances in patients with systemic inflammation	Hypertriglyceridemia, elevated low-density lipoprotein cholesterol (LDL-C), reduced high-density lipoprotein cholesterol (HDL-C)	[62]
Non-alcoholic fatty liver disease (NAFLD)	High free sugar intake is associated with an increased risk of NAFLD, mediated by fructose metabolism, which promotes de novo lipogenesis, inhibits fatty acid β-oxidation, and contributes to necroinflammation	Hepatic steatosis, elevated alanine aminotransferase (ALT) activity, hepatic fat accumulation	[63]
Cardiovascular disease	High intakes of total sugars, fructose, and added sugars are associated with an increased risk of cardiovascular mortality above certain intake thresholds	Coronary artery disease, myocardial infarction, stroke, cardiovascular mortality	[64]

In this context, biotechnological food reformulation may be regarded as a technologically flexible approach that enables modification of the carbohydrate composition of the food matrix without the use of synthetic additives.

One of the central strategies is the production of rare sugars, low-energy monosaccharides with a sweetness comparable to that of sucrose. These include D-allulose, D-mannose, D-sorbose, L-glucose, L-arabinose, and D-xylose. However, the regulatory status of individual rare sugars varies considerably across countries. In particular, D-allulose is regarded in several countries as a promising low-energy sugar substitute whose physiological effects have been demonstrated mainly in animal studies and short-term human studies, whereas in the European Union its use requires separate regulatory authorization [65,66]. Enzymatic biosynthesis of D-allulose using D-allulose 3-epimerase enables efficient bioconversion of readily available carbohydrate substrates (fructose, inulin, or sucrose-containing hydrolysates) with both immobilized enzymes and whole-cell microbial biocatalysts, including *Pseudomonas cichorii*, *Rhodobacter sphaeroides*, and *Clostridium scindens* [67]. Another important compound is D-tagatose, a naturally occurring epimer of D-fructose characterized by low metabolizable energy and minimal glycemic and insulinemic responses, owing to its limited absorption in the small intestine and partial fermentation by the gut microbiota [68]. Industrial biotechnological production of D-tagatose is based on the enzymatic isomerization of D-galactose by L-arabinose isomerase, which provides high product yields [69]. Other rare sugars, such as D-mannose, D-sorbose, L-glucose, L-arabinose, and D-xylose, are synthesized using various classes of immobilized biocatalysts (epimerases, isomerases, oxidoreductases, and transferases), thereby expanding the range of ingredients available for sugar reduction in mass-market food products [70,71]. Several of these compounds have been reported to affect metabolic processes, including inhibition of lipogenesis, activation of AMPK signaling, and enhancement of peripheral insulin sensitivity [72]. However, these mechanisms have been established mainly in cell models and animal studies, whereas in humans, primarily short-term effects on postprandial glycemia have been confirmed.

Another strategically important direction in the enzymatic reformulation of the carbohydrate composition of foods is the controlled synthesis of functional oligosaccharides, namely fructooligosaccharides (FOSs) and galactooligosaccharides (GOSs). Owing to their low glycemic index and pronounced prebiotic activity, they may modulate the gut microbiota and metabolic parameters associated with MetS. This approach is implemented through the enzymatic processing of agro-industrial waste using immobilized enzymes (β-fructofuranosidases, β-galactosidases) or whole microbial cells that enable the targeted synthesis of oligosaccharides [73].

In vitro studies, including a short-term colonic fermentation model [74] and an assessment of prebiotic potential using bacterial cultures [75], indicate that FOSs and GOSs produced by these enzymatic processes can selectively stimulate the growth of members of the genera *Bifidobacterium* and *Lactobacillus*. This was accompanied by increased SCFA concentrations and decreased levels of branched-chain fatty acids, pH, and ammonia in the fermentation medium. These changes are considered potentially beneficial to the host; however, host metabolic effects were not assessed in these models.

Alongside the strategies discussed above, a promising approach to reformulating carbohydrate composition is the use of resistant carbohydrates, including resistant starch (RS1-RS4), resistant dextrins, and slowly digestible carbohydrates. Their incorporation into food products is regarded as a potential approach to modulating metabolic disorders associated with MetS. Their possible metabolic effects are attributed to delayed glucose availability, altered substrate supply for energy metabolism, appetite modulation, and prebiotic effects mediated by colonic fermentation. However, the evidence for these effects is heterogeneous and comprises studies of varying design and level of evidence. In an animal study using a model of insulin resistance induced by a high-fat, high-fructose diet, resistant dextrin intake attenuated insulin resistance, suggesting potential metabolic benefits [76]. Human intervention data come from a randomized, single-blind, placebo-controlled trial in 20 individuals with insulin resistance and MetS, in which resistant starch consumption at 40 g/day for 12 weeks improved insulin sensitivity, as assessed by the hyperinsulinemic–euglycemic clamp, without significant changes in body weight or ectopic fat content [77]. Because the participants had MetS at baseline, these data pertain to a therapeutic context and do not allow conclusions to be drawn regarding a preventive effect. Observational data have more limited interpretive value: in a cross-sectional study, higher resistant starch intake was inversely associated with obesity in men, whereas no statistically significant association with MetS was found in women [78]. Fermentation of these compounds by the distal gut microbiota results in the production of SCFAs and changes in gut microbiota composition, which may contribute to improved metabolic homeostasis, including insulin sensitivity and lipid metabolism [79].

The metabolic efficacy of enzymatic strategies for reducing free sugars is determined not only by the chemical nature of the sucrose substitutes used, including rare sugars, functional oligosaccharides, and resistant carbohydrates, but also by the structural and functional characteristics of the food matrix. Product viscosity, the degree of starch hydration, and the content of dietary fiber, proteins, and lipids can substantially modulate the rate of release of available sugars, the extent of enzymatic hydrolysis, and the postprandial glycemic response. It is therefore important not only to reduce the absolute content of free sugars but also to design a product structure that slows starch digestion and the release of available sugars during digestion, increases the fermentability of non-digestible fractions, and enhances SCFA production by the gut microbiota. Given the above, the efficacy of carbohydrate reformulation should be assessed not only at the level of individual ingredients but also within specific food matrices that reflect real-life conditions of consumption and digestion.

### 4.2. Strategies for Reducing Salt Content in Food Products

Salt intake is a biological necessity, deeply integrated into the body’s physiological systems and historically rooted in food traditions worldwide. However, excessive salt intake is associated with elevated blood pressure and is estimated to cause 5 million deaths globally each year [80]. Beyond its direct effects on hemodynamic parameters, epidemiological evidence indicates a close association between sodium intake and the clustering of cardiometabolic risk factors. In particular, observational clinical studies have shown that patients with MetS exhibit significantly higher urinary sodium excretion (UNa), an objective marker of sodium intake [81]. In this population, elevated UNa levels are independently associated with obesity and arterial hypertension [82]. These observational data suggest that high sodium intake is associated not only with arterial hypertension but also with a broader spectrum of metabolic disorders; however, a causal relationship has not been established.

A key barrier to individual control of salt intake is the presence of “hidden” salt in industrially processed foods, which makes sodium reduction at the food industry level one of the priority population-level approaches [83]. Among conventional approaches to nutrient reformulation, partial replacement of NaCl with KCl, which has functional and technological properties similar to those of table salt, is the most widely used. However, studies have shown that direct replacement of NaCl with KCl is associated with undesirable sensory effects, the most significant of which are pronounced off-tastes, including bitterness, harshness, and a metallic aftertaste [84].

In this context, biotechnological approaches to food reformulation aimed at sodium reduction have attracted particular attention. This area is of additional relevance to MetS prevention because excessive salt intake, beyond its well-established association with arterial hypertension, is considered an independent risk factor for insulin resistance and impaired glucose homeostasis [85]. Key reformulation strategies aimed at preserving the sensory characteristics of food products include enzymatic modification of taste-active compounds and targeted modulation of taste–taste interactions, enabling sodium reduction without loss of perceived saltiness [86].

Most data on biotechnological strategies for sodium reduction have been obtained from technological and in vitro studies. Approaches based on the enzymatic hydrolysis of protein raw materials are of particular interest. As reported in a review, proteolysis of food proteins leads to the accumulation of low-molecular-weight peptides, predominantly di- and tripeptides, which act synergistically with Na^+^ ions [87]. In a sensory evaluation, peptides from an enzymatic pea protein hydrolysate enhanced perceived saltiness by approximately 20% in a 0.4% NaCl solution [88]. The possible involvement of the epithelial sodium channel (ENaC) in this effect remains hypothetical and is discussed on the basis of physiological data on the proteolytic regulation of ENaC [89]. Similarly, in a sensory evaluation, straw mushroom peptide hydrolysates containing low-molecular-weight fractions of 0.5–2.0 kDa increased the saltiness of a 0.05% NaCl solution to a level equivalent to that of a 0.15% solution; the involvement of the umami receptor T1R1/T1R3 in this effect was investigated in model experiments [90]. Soy protein hydrolysates containing peptides with molecular weights below 10 kDa are thought to modulate salty taste perception by binding to transmembrane channel-like 4 (TMC4). The peptides were fractionated by ultrafiltration and identified by tandem mass spectrometry, and their binding to TMC4 was predicted by in silico molecular docking [91]. In a technological study with sensory evaluation, enzymatic hydrolysis of pea protein with a combination of proteases and glutaminases, followed by extrusion, yielded a textured hydrolysate that significantly enhanced saltiness and umami taste at 0.5% NaCl. This enabled a 20% reduction in salt content in plant-based meat analog patties without the use of other additives [92].

Alongside enzymatic hydrolysis, an alternative biotechnological approach involves the use of microbial cultures for the biotransformation of taste-active precursors. In particular, fermentation of food matrices with strains of *Tetragenococcus halophilus* and *Planococcus maritimus*, in combination with the yeasts *Zygosaccharomyces rouxii* and *Candida versatilis*, promotes the accumulation of organic acids, free amino acids, and short-chain esters, resulting in a complex flavor profile in reduced-sodium seasonings, fish sauce, and soy sauce [93,94]. In the context of MetS prevention, this approach may offer an additional advantage: bioactive compounds with antihypertensive, antioxidant, and anti-inflammatory activities that accumulate during fermentation have exhibited antidiabetic activity in experimental systems [95]. However, such effects have been characterized mainly in technological studies and in vitro experiments and have not been evaluated in humans.

The use of nucleotides and their derivatives, such as guanosine 5′-monophosphate (GMP) and inosine 5′-monophosphate (IMP), is a promising strategy for reducing sodium chloride content in foods. Sensory studies have shown that GMP acts in pronounced synergy with monosodium glutamate and sodium ions, resulting in enhanced salty taste [96]. Molecular modeling data suggest that this effect is due to allosteric modulation of the heterodimeric T1R1/T1R3 receptor, mediated by ligand interaction with the ligand-binding domain of the receptor complex [97]. These nucleotides are produced biotechnologically by microbial fermentation using producer strains of the genera *Corynebacterium*, *Bacillus*, or *Escherichia*, which enables sustainable and cost-effective production of flavor enhancers for industrial reformulation [98].

It should be noted that the most effective salt reduction strategies combine several approaches within a single formulation strategy. For example, a technological study showed that replacing 50% of NaCl with KCl, combined with the addition of lysine and a mixture of 5′-ribonucleotides (IMP and GMP) with taurine, made it possible to produce sausages with high sensory quality and a substantially reduced sodium content [99]. Data from a cluster randomized trial in humans using reduced-salt bread indicate that the consumption of reduced-salt reformulated products leads to a statistically significant decrease in 24-h urinary sodium excretion, which is regarded as an objective biomarker of reduced dietary sodium intake [100].

Saltiness perception is determined by the distribution of sodium ions within the food matrix, moisture content, porosity, particle size, rheological properties, water-binding capacity, and the protein–fat structure of the product. Integrating a matrix-based approach allows salt reduction to be viewed not only as a nutrient adjustment but also as a matter of controlling the spatial distribution and release kinetics of taste-active compounds. Therefore, combined approaches in which sodium reduction is accompanied by optimization of the matrix structure, enhancement of taste perception, and maintenance of consumer acceptance appear to be the most promising. This strategy is particularly important for mass-market foods, as it is the food matrix that determines the feasibility of sodium reduction, sensory acceptability, and relevance for the prevention of arterial hypertension and other components of MetS.

### 4.3. Biotechnological Optimization of the Lipid Profile

Lipid metabolism disorders play a central role in the pathogenesis of MetS, acting both as an initiating factor of the pathological process and as a reliable predictor of adverse cardiovascular outcomes [101]. Qualitative changes in the lipoprotein profile are of particular prognostic importance. In particular, individuals with obesity often exhibit atherogenic dyslipidemia, characterized by elevated levels of small, dense low-density lipoprotein (sdLDL) particles, triglycerides (TGs), very-low-density lipoprotein (VLDL), and apolipoprotein B, along with a reduced concentration of high-density lipoprotein cholesterol (HDL-C) [102].

One of the most important dietary factors contributing to this adverse lipid profile is excessive intake of saturated fatty acids (SFAs) and trans fatty acids (TFAs). Accumulated evidence indicates that SFAs alter lipid metabolism by slowing the clearance of LDL apolipoprotein B-100 (apoB-100) and apolipoprotein A-I (apoA-I) from the circulation, which contributes to the development of an atherogenic phenotype, while simultaneously increasing apoA-I production [103]. Dietary TFAs, in turn, trigger a cascade of metabolic disturbances, including adverse changes in the lipid profile, induction of low-grade systemic inflammation, and the development of endothelial dysfunction [104]. Furthermore, findings from observational and experimental studies, summarized in reviews, indicate that TFA intake is associated with visceral fat accumulation, weight gain, and the progression of insulin resistance, which exacerbates dyslipidemia and complements the effects of excess SFA intake [105,106].

Given the established pathophysiological role of excess SFAs and TFAs in the onset and progression of metabolic disorders, a primary objective of biotechnological food reformulation is to reduce the proportion of these components while preserving the sensory and textural properties of the final product. Since conventional approaches to reducing solid fat content are limited by technological requirements, enzymatic interesterification has emerged as a relevant approach for the targeted modification of the triacylglycerol profile without the formation of trans isomers. This process, catalyzed by various types of lipases, involves the rearrangement of fatty acids on the glycerol backbone, resulting in structured lipids with altered physicochemical and metabolic properties [107].

**Figure 2:**
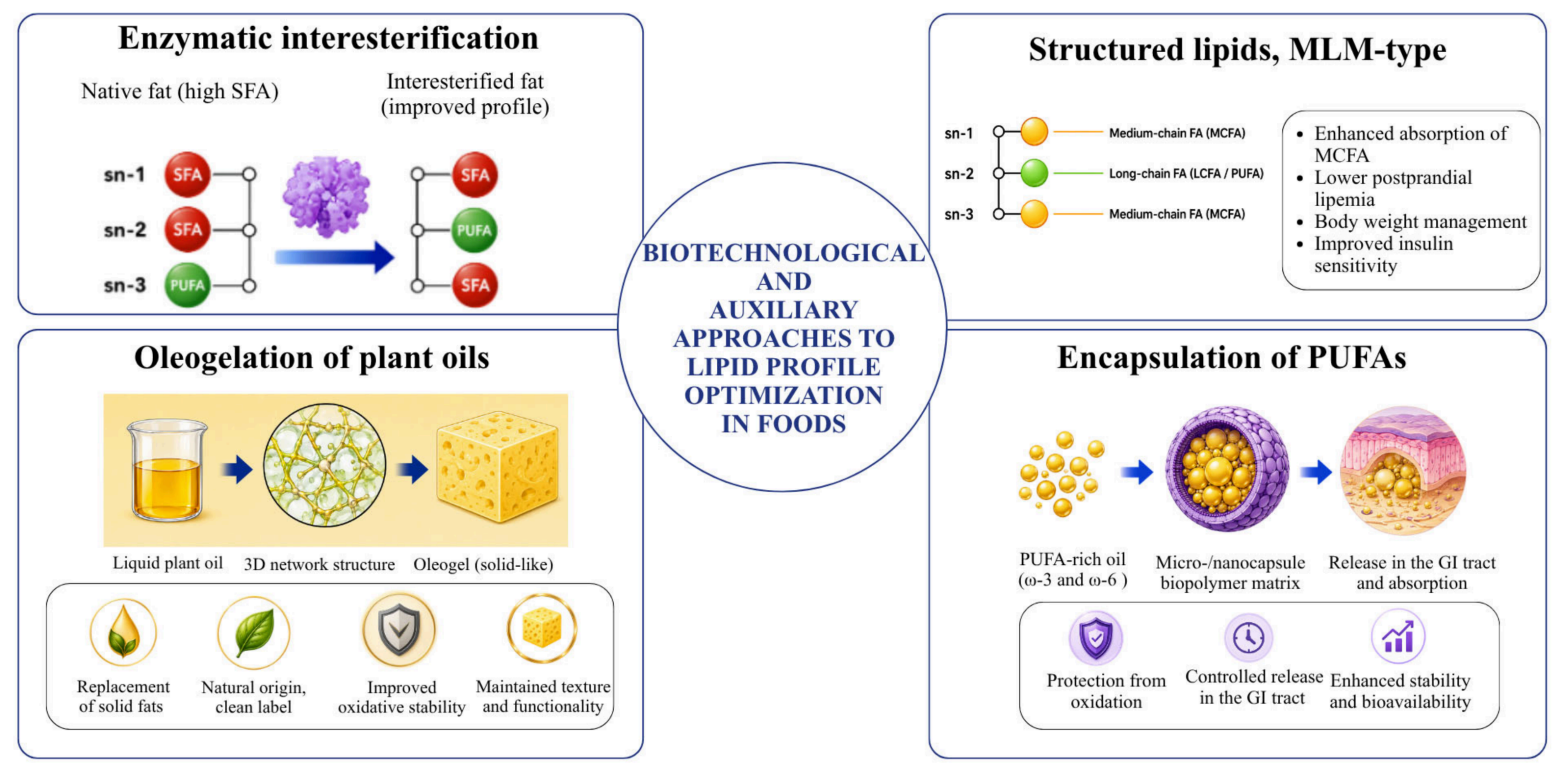
Conceptual representation of biotechnological and supporting technological approaches for lipid profile optimization in foods. The scheme illustrates potential approaches for modifying the lipid composition and physicochemical properties of food matrices through enzymatic interesterification, lipid structuring, oleogelation, and PUFA encapsulation. The depicted technological approaches and associated physiological effects reflect evidence obtained at different levels and should not be interpreted as a continuous or universally established causal pathway. The indicated metabolic and functional effects represent potential or literature-described consequences of corresponding modifications of the lipid matrix and do not constitute independent evidence of the effectiveness of reformulated foods for MetS prevention. In particular, physiological effects reported for individual lipid components or structured lipid systems should not be directly extrapolated to clinical effects of reformulated foods. Oleogelation and encapsulation are presented as auxiliary technological tools for structuring and stabilizing lipid components within the food matrix, rather than as standalone biotechnological strategies for MetS prevention.

Structured lipids enriched in medium-chain fatty acids (MCFAs) are of particular interest for MetS prevention because, unlike long-chain fatty acids (LCFAs), MCFAs undergo rapid hepatic oxidation and accumulate in adipose tissue to a much lesser extent. The use of sn-1,3-specific lipases enables the production of medium–long–medium (MLM)-type structured lipids with a strictly defined positional distribution: MCFAs (C6–C12) are located predominantly at the sn-1 and sn-3 positions of the glycerol backbone, whereas the sn-2 position is occupied by LCFAs (C14–C24). Current biocatalytic strategies, including the use of heterologously expressed recombinant lipases derived from fungi of the genera *Rhizopus* and *Thermomyces* and from bacteria of the genera *Pseudomonas* and *Burkholderia*, enable efficient position-specific rearrangement of fatty acids in triacylglycerols to yield MLM-type structured lipids, while improving the operational stability of the biocatalyst and the environmental sustainability of the process [108,109,110]. MLM lipids have a reduced energy value and are of interest as an experimental approach to modifying the lipid profile of food products. Data on their potential metabolic effects on excess body weight, lipid absorption, and cardiometabolic disorders are largely based on technological studies, mechanistic research, and preclinical models, whereas clinical evidence remains limited [111]. In a preclinical study in ApoE−/− mice fed a high-fat diet, MLM lipid consumption was associated with a reduced atherosclerotic lesion area, decreased lipid accumulation in adipose tissue, and favorable modulation of gut microbiota composition, reflected in an increased abundance of SCFA-producing bacteria [112]. On an industrial scale, enzymatic interesterification is already used to produce modified fats, including TFA-free margarines and shortenings, cocoa butter equivalents, human milk fat substitutes, and low-calorie lipid components [113]. Biotechnological and auxiliary (non-biotechnological) technological approaches to optimizing the lipid profile of food products are presented in Figure 2.

An alternative approach to eliminating solid animal fats high in SFAs is to replace them with vegetable oils, which are sources of PUFAs. However, the direct incorporation of oils into emulsion-based and confectionery formulations leads to structural deterioration, oil leakage, and a marked decrease in oxidative stability. This problem can be addressed by oleogelation, a technology that structures liquid oils and thereby improves the nutritional profile of food products while preserving their technological functionality [114]. Oleogelation is not a biotechnological process and is considered in this review as an auxiliary technology, including for the structuring of oils of biotechnological origin (microbial oils and enzymatically produced structured lipids). Oleogels are produced by entrapping the liquid oil phase within a continuous three-dimensional network formed by low-molecular-weight oleogelators (LMOGs) or high-molecular-weight oleogelators (HMOGs) [115]. Unlike chemical interesterification, this approach not only eliminates SFAs and TFAs from the formulation through the physical immobilization of vegetable oils but may also modulate postprandial lipemic and glycemic responses (according to a limited number of acute human studies) and provide protection of thermolabile bioactive compounds against oxidative degradation within the food matrix [116]. Technological and sensory studies have demonstrated the feasibility of producing reformulated products, including spreads, bakery and confectionery products, and dairy and meat products, with technological and sensory characteristics comparable or superior to those of conventional counterparts and a substantially improved fatty acid profile [117].

The incorporation of PUFAs into reformulated products is supported by evidence linking them to key pathogenic mechanisms of MetS [118], but it is limited by their oxidative instability. Encapsulation improves the oxidative stability of PUFAs during processing and storage and enables their retention in functional food products. Methods such as spray-drying microencapsulation, liposomal vesicles, and multilayer emulsion systems provide effective protection of PUFAs against oxidative degradation during storage and passage through the gastrointestinal tract [119]. A study in individuals with an ileostomy showed that PUFAs from encapsulated fish oil incorporated into various food matrices reach the ileum [120]; however, the physiological relevance of this finding for MetS components has not been established.

An analysis of biotechnological and auxiliary technological approaches to modifying the lipid profile of food products demonstrates their considerable potential for the development of reformulated food matrices. However, evidence on the clinical efficacy of these approaches against atherogenic dyslipidemia in MetS remains heterogeneous. While the efficacy of some strategies has been confirmed in controlled human studies, the evidence for others, particularly MLM lipids and several encapsulated lipid systems, is largely limited to mechanistic and animal studies.

### 4.4. Protein Reformulation and Appetite Regulation

Current evidence indicates that high-protein diets are associated with greater weight loss and improvements in cardiovascular health parameters compared with standard-protein diets [121]. Such dietary approaches are currently regarded as a potential strategy for the prevention of obesity, MetS, and associated impairments in glycemic regulation. Given the central role of the gut–brain axis in the regulation of energy homeostasis, modification of the protein component of food products represents a promising strategy for enhancing their nutritional value, one that may also influence the postprandial hormonal response and satiety [122]. Biotechnological transformation of protein matrices, including enzymatic hydrolysis and targeted modification of native protein structure, makes it possible to regulate digestion kinetics and, consequently, the secretion of hormones that control satiety and suppress appetite [123].

Conventional strategies for increasing dietary protein content rely on protein isolates, concentrates, and hydrolysates of both animal (whey and casein) and plant (soy and pea) origin [124,125,126]. These sources differ substantially in amino acid absorption kinetics and in their ability to stimulate incretin secretion. Whey protein is characterized by rapid digestion and a high leucine content, resulting in a rapid postprandial rise in plasma insulin, amino acids, GIP, and GLP-1 [127]. In contrast to whey, casein coagulates in the acidic gastric environment to form a dense curd. This structural transformation results in a slower amino acid release profile, contributing to prolonged satiety [128]. Thus, differences in the physicochemical properties and hydrolysis rates of protein components account for the variability in postprandial metabolic and hormonal responses, which supports the development and implementation of purposefully modified protein ingredients with tailored functional properties.

One of the key biotechnological approaches to protein modification is targeted enzymatic hydrolysis using specific endo- and exopeptidases to produce bioactive peptides and hydrolysates. According to a review of predominantly preclinical animal studies, these compounds may selectively modulate gut microbiota composition by suppressing the proliferation of obesity-associated taxa and promoting the growth of microorganisms associated with a “lean” phenotype, accompanied by downregulation of pro-inflammatory transcription factors [129]. A review of in vitro, preclinical, and limited clinical evidence indicates that endogenous and exogenous peptides of various origins containing up to 20 amino acid residues exhibit anti-obesity properties, demonstrated predominantly in vitro and in preclinical models [130]. In particular, whey protein hydrolysates containing bioactive peptides can stimulate the secretion of several gut hormones, including cholecystokinin, peptide YY (PYY), and the incretins glucose-dependent insulinotropic polypeptide (GIP) and glucagon-like peptide-1 (GLP-1), which enhance insulin release from β-cells and are involved in the regulation of food intake; these effects are potentially relevant to MetS components [131].

In addition to peptide structures acting on specific receptors, individual amino acids may also exert beneficial effects in metabolic disorders. In particular, arginine, leucine, glycine, and glutamine are involved in the modulation of mTOR signaling and nitric oxide (NO)-dependent mechanisms; however, these data have been obtained predominantly with isolated amino acid supplementation rather than with amino acids consumed as part of food products [132].

An alternative approach to modifying the functional properties and improving the digestibility of proteins is biotechnological processing using microorganisms—particularly lactic acid bacteria, fungi of the genera *Aspergillus* and *Rhizopus*, and yeasts of the genera *Saccharomyces* and *Candida* [133]. This process involves partial proteolysis, biotransformation of amino acids, and accumulation of aroma compounds, which improves the sensory properties of reformulated products. In a randomized, double-blind, placebo-controlled trial involving 197 patients with MetS, consumption of low-fat yogurt enriched with the fermented whey product known as malleable protein matrix (MPM) for 12 weeks was associated with a statistically significant reduction in fasting plasma TG concentrations, as well as favorable changes in several other cardiovascular risk factors [134]. This study was conducted in patients with diagnosed MetS and therefore characterizes the therapeutic rather than the preventive potential of the product.

Matrix-dependent mechanisms of protein reformulation determine the rate of protein digestion, the release of amino acids and bioactive peptides, and the postprandial hormonal response. The physiological effects of protein isolates, concentrates, hydrolysates, and fermented protein ingredients depend not only on their amino acid composition and degree of hydrolysis but also on the physical form of the product, its viscosity, gelling capacity, and interactions with the macro- and micronutrients of the final product. Liquid protein products, soft gel systems, solid bars, fermented dairy products, and plant-based protein matrices may differ substantially in the rate of gastric emptying, substrate accessibility to proteases, and the magnitude of GLP-1, GIP, PYY, and cholecystokinin secretion. Consequently, the potential of protein reformulation for the prevention of obesity and MetS depends not only on increasing protein content but also on designing food structures with a defined rate of digestion, predictable peptide release during digestion, and reproducible effects on appetite, satiety, and metabolic markers.

### 4.5. Strategies for Improving Micronutrient Bioavailability and Stability

Nutrient status is regarded as one of the factors associated with the pathogenesis of MetS, since deficiencies in certain vitamins and trace elements may affect oxidative stress, mitochondrial dysfunction, and systemic inflammation, which are key mechanisms underlying this condition. Fortification of mass-market food products with essential micronutrients, including through biotechnological approaches (fermentation and biofortification), may be regarded as a physiologically sound approach to maintaining metabolic health at the population level. The efficacy of this approach is being investigated in randomized trials; in particular, a protocol has been published for a study of zinc-enriched wheat flour subjected to fermentation [135]. Effects of fortified products on metabolic parameters have been demonstrated in animal models [136]; however, their extrapolation to humans requires confirmation in controlled clinical trials. According to recent studies, functional foods and dietary supplements may improve insulin sensitivity and glucose tolerance in individuals with metabolic and gastrointestinal disorders [137]; however, these data pertain predominantly to a therapeutic context. In addition, improved bioavailability of essential nutrients may be associated with favorable changes in certain markers of obesity, arterial hypertension, and dyslipidemia; however, the clinical relevance of these effects requires further confirmation [138].

This approach was investigated in the PATHWAY-27 pilot study conducted in adults with MetS risk factors, which showed that consumption of foods enriched with bioactive compounds improved lipid metabolism parameters, including MetS markers [139]. The efficacy of the intervention depended not only on the dose and type of compounds used but also on the carrier food matrix: dairy products enriched with DHA and natural polysaccharides significantly reduced TG levels and increased HDL-C concentrations, with no serious adverse events. A study in rats with high-fat diet-induced MetS likewise showed that a functional fermented product enriched with carotenoids and phytosterols attenuated several manifestations of MetS in the animals, reducing adipose tissue mass and improving the lipid profile, vitamin A status, and glucose tolerance [140].

However, one of the key limitations reducing the effectiveness of direct fortification is the bioavailability of the added nutrients, which is compromised by their chemical instability within the complex food matrix and the harsh environment of the gastrointestinal tract, as well as by their potential undesirable interactions with other components of the food system [141]. In response to these challenges, fortification approaches have shifted from the simple quantitative addition of nutrients toward the development of fermented (a biotechnological approach) and encapsulated (an auxiliary non-biotechnological technology) nutrient forms with enhanced stability in the food matrix and improved bioavailability.

Fermentation-based approaches to food reformulation enhance the nutritional and functional value of foods by increasing mineral bioavailability and generating bioactive compounds. For example, according to compositional analysis, fermentation of soy milk with the lactic acid bacteria strains *Lactobacillus fermentum* B4655, *L. plantarum* B4495, *L. casei* B1922, *L. bulgaricus* CFR2028, and *L. acidophilus* B4496 increased Mg and Ca concentrations in the fermented product, accompanied by a decrease in phytic acid content [142]. Similar patterns of enhanced mineral bioavailability (Ca, Na, Cu, Fe, Zn, and K) resulting from microbial phytase activity have been reported for a wide range of cereal crops, including millet [143]. In addition to minerals, fermentation enhances vitamin bioavailability. A review of compositional studies showed that, in most of the studies examined, fermentation of maize, buckwheat, rice, and sorghum with various starter cultures (lactic acid bacteria, yeasts, and fungi) was accompanied by increases in vitamins B1, B2, B3, and E of up to tenfold [144]. These fermentation processes may be regarded as a biotechnological method of endogenous enrichment in product reformulation; however, the effect of such products on MetS risk has not been studied.

Unlike fermentation, encapsulation does not alter the composition of bioactive compounds; rather, it protects them from degradation during processing and storage, enhances their stability in the gastrointestinal tract, and masks undesirable sensory properties, thereby improving the acceptability of reformulated products [145,146]. Encapsulated forms of polyphenols, probiotics, prebiotics, fatty acids, and vitamins have been described in reviews [147,148] and animal studies [149]; in a human study, the encapsulated component was administered as a supplement rather than as part of a food product [150].

Thus, fermentation and encapsulation may increase the content, stability, and bioavailability of micronutrients in mass-market food products. To assess the practical value of these approaches, it is necessary to determine which food matrix–ingredient combinations ensure nutrient stability during processing and storage, acceptable sensory properties, and bioavailability, and to conduct long-term human studies comparing reformulated products with their conventional counterparts. Table 2 summarizes biotechnological strategies for food reformulation, together with the associated auxiliary technologies, targeting key MetS components.

The studies summarized in Table 2 have several limitations. The in vitro studies were based mainly on cell-free assays that do not reflect gastrointestinal digestion, absorption, or bioavailability of the bioactive compounds. The animal studies used induced or genetic models that reproduce only individual components of MetS, applied doses exceeding realistic dietary intake, in some cases administered the test material outside a food matrix, and had relatively short intervention periods. Human studies were predominantly acute postprandial crossover trials with small sample sizes, conducted mainly in healthy volunteers rather than in individuals with MetS; two studies were cross-sectional and therefore did not permit causal inferences. In most intervention studies, the control condition did not include a non-reformulated counterpart of the same product, so the contribution of the biotechnological modification could not be separated from that of the food matrix. In addition, the studies relied on surrogate biomarkers and did not assess long-term clinical outcomes, and the heterogeneity of interventions and endpoints limited comparison between them.

## 5. Bioactive Compounds as Potential Ingredients for Food Reformulation

### 5.1. Probiotics and Synbiotics

The gut microbiota is currently the subject of extensive research owing to its multifaceted influence on health, encompassing a wide range of physiological and pathological functions. The composition of the microbial community is shaped by bidirectional interactions with the host, and a shift in this balance toward dysbiosis may, according to predominantly experimental and observational studies, contribute to the pathogenesis of MetS. In particular, a dysbiotic microbiome may promote the activation of pro-inflammatory pathways characteristic of obesity phenotypes. These include increased absorption of lipopolysaccharides from Gram-negative bacteria and the translocation of endotoxins into the systemic circulation due to impaired intestinal barrier permeability [170]. Animal studies suggest that the obesity-associated microbiota may have an increased capacity to harvest energy from non-digestible carbohydrates, including through the production of SCFAs that serve as substrates for lipogenesis [171]. Taken together, these mechanisms, described mainly in experimental studies, provide a rationale for interest in probiotics as a tool for modulating the gut microbiota [172,173].

#### 5.1.1. Mechanistic Evidence

Probiotic interventions—particularly those using *Bifidobacterium* and *Lactobacillus* strains—and synbiotics combining probiotic strains with prebiotic components may influence metabolic function. Their proposed mechanisms of action include modulation of gut microbiota composition, regulation of the microbial metabolite profile, and improvement of intestinal barrier function [174].

#### 5.1.2. Animal Studies

In a study in rats with MetS induced by a high-fructose diet, administration of a mixture of the probiotic strains *Lactobacillus curvatus* HY7601 and *Lactobacillus plantarum* KY1032 at both a high dose (10^10^ CFU/day) and a low dose (10^9^ CFU/day) was accompanied by reductions in plasma concentrations of glucose, insulin, TGs, and oxidative stress markers. The high dose additionally reduced liver weight and hepatic cholesterol content [175]. In Wistar rats with high-fat diet-induced obesity, the effects of a synbiotic comprising the probiotic strain *Lactobacillus paracasei* HII01 (10^8^ CFU/mL) and the prebiotic xylooligosaccharide (XOS, 10%) were investigated [176]. Both the individual components and their combination were equally associated with attenuation of gut dysbiosis and inflammation, an improved lipid profile, and increased insulin sensitivity. These findings support the biological plausibility of metabolic effects; however, their translation to humans is limited by the specific features of animal models.

#### 5.1.3. Human Studies

Human intervention studies have been conducted mainly with probiotics and synbiotics administered as dietary supplements in individuals with existing disorders of carbohydrate metabolism, and their results are variable. In individuals with prediabetes, beneficial effects on glycemic parameters have been reported for a consortium of the probiotic strains *Lactobacillus acidophilus*, *Bifidobacterium lactis*, and *Bifidobacterium bifidum* (10^9^ CFU each), as well as for a synbiotic based on these strains combined with inulin [177]. The synbiotic was associated with a more pronounced reduction in fasting plasma glucose, fasting insulin, and the homeostasis model assessment of insulin resistance (HOMA-IR) than the probiotic alone, as well as a significant increase in the quantitative insulin sensitivity check index (QUICKI). Similar results were obtained in patients with type 2 diabetes mellitus in a study of the multistrain probiotic Symbiter, a concentrated biomass of 14 probiotic bacteria belonging to the genera *Bifidobacterium*, *Lactobacillus*, *Lactococcus*, and *Propionibacterium* [178]. Its administration was associated with significant reductions in HOMA-IR, HbA1c, and concentrations of the pro-inflammatory cytokines TNF-α and IL-1β. In another study in patients with prediabetes, the synbiotic Lactocare was associated with significant reductions in body weight, fasting plasma glucose, HbA1c, triglycerides, and LDL-C, as well as increases in insulin and HDL-C levels [179]. This synbiotic contains the probiotic strains *Lactobacillus casei*, *Lactobacillus acidophilus*, *Lactobacillus rhamnosus*, *Lactobacillus bulgaricus*, *Bifidobacterium breve*, *Bifidobacterium longum*, and *Streptococcus thermophilus*, together with the prebiotic fructooligosaccharide.

Thus, animal studies support the biological plausibility of the effects of probiotics and synbiotics on glycemic and lipid parameters and on gut microbiota composition. Intervention studies in individuals with prediabetes and patients with type 2 diabetes mellitus suggest that modest improvements in certain cardiometabolic markers are possible. Because these studies were conducted with dietary supplements in individuals with existing disorders, their results do not constitute evidence of MetS prevention. Optimal strains, doses, and food matrices, as well as the efficacy of probiotics and synbiotics incorporated into reformulated foods, require further investigation.

### 5.2. Dietary Fibers and Prebiotics

Dietary fiber and prebiotics are attracting increasing attention as key nutritional modulators of gut microbiota and metabolic homeostasis in MetS. Many dietary fiber and prebiotic compounds escape digestion in the upper gastrointestinal tract and reach the colon intact, where they directly interact with gut microorganisms. Through this interaction, they exert prebiotic effects by modulating the composition of microbial communities and enhancing the production of bioactive metabolites, particularly SCFAs [180,181]. By selectively stimulating the growth and activity of SCFA-producing bacteria, these bioactive compounds may, according to predominantly experimental studies, help maintain intestinal barrier integrity, reduce metabolic endotoxemia and inflammation, and improve insulin sensitivity [182].

Current studies indicate an association between increased dietary fiber intake and decreased fat mass, improved glycemic control, and lower blood pressure, TG, and LDL-C levels [183,184,185,186]. Combining different types of dietary fiber to optimize their physiological effects is of particular interest. It is believed that consuming a balanced fiber blend, aimed at reducing small intestinal transit time and prolonging colonic retention, is thought to slow nutrient absorption and thereby help control postprandial blood glucose [187].

#### 5.2.1. Animal Studies

In vivo experiments have demonstrated that different types of dietary fiber differ in their effectiveness for glycemic control: β-glucan, arabinogalactan, guar gum, apple pectin, glucomannan, and arabinoxylan significantly reduced fasting blood glucose levels in rats with induced type 2 diabetes mellitus, whereas carrageenan, xylan, and xanthan gum did not have a statistically significant effect [188]. These data emphasize that the metabolic effects of dietary fiber are not universal and depend on their structure, solubility, fermentability, and interaction with the food matrix.

The hypolipidemic properties of dietary fiber have also been reported. For example, in rats fed a high-fat diet, consumption of chickpea dietary fiber led to a significant decrease in the levels of total cholesterol, TG, and LDL-C, and an increase in the level of HDL-C [189]. Additionally, an increase in *Bacteroides* and *Lactobacillus* counts, as well as an increase in propionic acid levels, was observed, indicating a beneficial effect of these fibers on the serum lipid profile and intestinal microbiome.

#### 5.2.2. Human Studies

Clinical studies indicate that modulation of the gut microbiota by prebiotic fibers may affect inflammatory markers associated with SCFA production. In a pilot open-label randomized trial, consumption by patients with MetS of a prebiotic blend containing fructooligosaccharides (FOSs), inulin, resistant dextrin, resistant maltodextrin, partially hydrolyzed guar gum, and guar gum was associated with a significant reduction in high-sensitivity C-reactive protein (hs-CRP) levels, as well as with an increase in the fecal abundance of the SCFA producers *Bifidobacterium* and *Parabacteroides* [190]. However, because the study was conducted in patients with MetS, these data characterize the therapeutic potential of prebiotic blends and do not constitute evidence of MetS prevention.

In overweight adults with prediabetes, short-chain FOS supplementation was associated with favorable changes in gut microbiota composition and enzymatic activity, as well as modest improvements in body composition, including a decrease in fat mass and an increase in lean mass, a selective increase in *Bifidobacterium* and *Anaerostipes*, and an increase in fecal acetate and propionate levels, while maintaining stable total body weight [191]. Furthermore, a combination of rice bran and Jerusalem artichoke dietary fiber with rice anthocyanins improved glycemic control, the lipid profile, oxidative stress and inflammation markers, and cardiorespiratory fitness, reducing HbA1c and LDL-C levels and improving the glomerular filtration rate in patients with type 2 diabetes mellitus [192]. These studies were conducted in individuals with prediabetes and type 2 diabetes mellitus; therefore, their results are only indirectly applicable to MetS prevention.

Taken together, the data presented indicate that dietary fibers and prebiotics are promising ingredients for reformulated products, capable of influencing several MetS components, with their effects largely mediated by interactions with the gut microbiota. The heterogeneity of the physicochemical properties of different types of fiber determines their differential effects on glycemic control, lipid profile, and satiety, making it inappropriate to extrapolate the effects observed for one type of fiber to the entire group as a whole. Owing to their gel-forming capacity and fermentability in the colon, soluble dietary fibers may influence most MetS components, whereas insoluble fibers have been reported to affect only some of them through other mechanisms [193]. Therefore, further research should focus on clarifying the effects of fiber type and dose, the role of the food matrix, and the sustainability of clinical responses in long-term intervention studies.

### 5.3. Polyunsaturated Fatty Acids

The structure of PUFAs is relatively simple and consists of three elements: carbon, hydrogen, and oxygen. The key difference between individual PUFAs lies in the number and position of double bonds in the molecule. In ω-3 PUFAs, the first double bond, counting from the methyl end of the chain, is located at the third carbon atom, whereas in omega-6 PUFAs, it is located at the sixth [194]. Based on this structural criterion, two categories are distinguished: ω-3 and ω-6 polyunsaturated fatty acids.

When interpreting data on PUFAs, it is necessary to distinguish between two different types of exposure. The first is the isocaloric replacement of SFAs with PUFAs, in which the effect is determined simultaneously by reduced SFA intake and increased PUFA intake. The second is an increase in the intake of an individual fatty acid (e.g., linoleic acid or eicosapentaenoic acid (EPA)/DHA) without changes to the rest of the diet, including in the form of dietary supplements. The effects of these exposures are not equivalent, and results obtained for one cannot be extrapolated to the other. The first type of exposure is the most relevant to food reformulation, since modifying the fatty acid composition of a product typically means replacing some fatty acids with others.

Observational studies and their meta-analyses indicate that higher blood levels of omega-6 PUFAs, primarily linoleic acid, are associated with a lower risk of coronary heart disease [195,196]. According to a pooled analysis of prospective cohort studies using biomarkers, higher linoleic acid levels are also associated with a lower risk of type 2 diabetes mellitus [197]. These data reflect associations and do not allow the effect of omega-6 PUFAs per se to be distinguished from that of the dietary components they replace.

Omega-6 and ω-3 PUFAs compete for shared desaturation and elongation enzyme systems. On this basis, several reviews have considered the ω-6/ω-3 PUFA ratio: a higher ratio, that is, a relative excess of ω-6 over ω-3, is associated with obesity and chronic low-grade inflammation [198], as well as with impaired carbohydrate metabolism [199]. However, the interpretation of this indicator is limited. The ratio does not reflect the absolute intake of each PUFA group: the same value may correspond to either low or high intake of both groups. Moreover, it does not distinguish between plant-derived α-linolenic acid (ALA) and long-chain EPA and DHA, which differ in biological activity. Therefore, when evaluating reformulated products, it is advisable to consider the absolute content of individual PUFAs rather than only their ratio.

Prospective observational studies indicate that higher intake of ω-3 PUFAs, particularly ALA, EPA, and DHA, is associated with lower cardiometabolic risk [200,201]. Proposed mechanisms include reduced plasma TG concentrations, changes in lipoprotein particle size, suppression of inflammatory processes, atherosclerotic plaque stabilization, improved vascular function, and antiarrhythmic effects. In an intervention study in healthy older women, ω-3 PUFA supplementation for 12 weeks was accompanied by an increased metabolic rate at rest and during exercise, reduced fat mass, and increased lean body mass [202]. These effects are thought to be partly mediated by the ability of ω-3 PUFAs to act as ligands of peroxisome proliferator-activated receptors (PPARs), which regulate β-oxidation and glucose metabolism [203].

Since most of the data presented were obtained from observational studies or studies of isolated PUFA supplementation, further progress requires the evaluation of reformulated foods with a modified fatty acid composition, including those enriched with microbial oils. Such studies will help determine whether the observed associations and effects are reproduced when PUFAs are consumed within a food matrix and replace other dietary fatty acids.

### 5.4. Vitamins and Minerals

Vitamins and minerals play a key role in a wide range of cellular and biochemical processes, including energy metabolism, biosynthesis, and other physiological processes, acting as coenzymes, cocatalysts, and buffering compounds [204]. A growing body of evidence suggests that a wide range of metabolic disorders, including increased oxidative stress, insulin resistance, endothelial dysfunction, and impaired glucose and lipid metabolism, correlate with the phenomenon of “hidden hunger”—a subclinical deficiency of vitamins and minerals that develops despite excess energy intake [205,206,207]. This paradox may be partly attributable to the fact that highly refined foods associated with an increased risk of MetS are characterized by low micronutrient density.

Water- and fat-soluble vitamins are essential organic compounds required for normal growth and functioning of the body. Their absence from the diet inevitably leads to the development of various pathological conditions. In addition to their involvement in fundamental physiological processes, vitamins with antioxidant and anti-inflammatory activity are being investigated in relation to MetS components [208].

#### 5.4.1. Animal Studies

Experimental and clinical studies have investigated the effects of vitamins on MetS components. In particular, in vivo experiments in rats showed that administration of vitamins A, C, and E reduced serum levels of total cholesterol, TG, LDL-C, and VLDL-C, while increasing HDL-C levels and total antioxidant status, in animals fed a high-salt diet [209]. These findings suggest a potential effect of antioxidant vitamins on the lipid profile and oxidative stress under experimental conditions; however, their relevance to humans, particularly when delivered as part of food products, requires confirmation.

Furthermore, the effects of vitamin D on metabolic disorders have been examined in experimental models. In a recent study in rats fed a high-fat diet, vitamin D administration was associated with a significant attenuation of key MetS manifestations, including obesity, arterial hypertension, and dyslipidemia [210]. Another in vivo study demonstrated the ability of B vitamins to reduce body weight gain by improving the activity of enzymes associated with energy metabolism in rats fed a high-fat diet. The results of the study showed that daily administration of vitamins B1, B2, and niacin significantly limited body weight gain and reduced plasma TC and TG levels in animals, which was associated with an increase in the activity of transketolase, glutathione peroxidase, Na^+^/K^+^-ATPase, and other key energy metabolism enzymes [211].

#### 5.4.2. Human Studies

These experimental data are consistent with the results of clinical studies that have examined the effects of vitamin supplementation on MetS components. For example, a study in adult patients with MetS demonstrated that the combination of daily vitamin C supplementation with moderate physical activity can significantly reduce body mass index and total serum cholesterol levels, as well as have a positive effect on systolic blood pressure [212]. In this case, the effect should be considered as the result of a combined intervention since the contribution of vitamin C cannot be fully separated from the effect of physical activity. Similar beneficial effects on MetS components, including improved lipid profiles, reductions in blood pressure and inflammatory markers, have been reported with B vitamins [213], vitamin D [214], vitamin E [215] and vitamin K [216]. It should be noted that these studies used dietary supplements in patients with MetS or coronary artery disease, or in individuals with overweight and obesity, and therefore primarily reflect a therapeutic context. However, clinical findings are not completely uniform, and the magnitude of the effect may vary considerably depending on the micronutrient status of the participants. Therefore, the most justified approach to reformulation appears to be the fortification of mass-market food products with those micronutrients whose deficiency is prevalent in the population.

Along with vitamins, minerals play a significant role in the regulation of metabolic processes in MetS. Their biological activity is realized through participation in insulin-dependent signaling cascades, regulation of carbohydrate and lipid metabolism, as well as maintaining the antioxidant status of the body [217,218]. Epidemiological data indicate a high prevalence of magnesium, zinc, and chromium deficiency among individuals with MetS, which correlates with worsening insulin resistance, dyslipidemia, and hypertension [219,220,221].

Randomized supplementation trials have shown that restoring adequate micronutrient status may also contribute to improvements in individual MetS components. In particular, magnesium intake has been associated with reductions in fasting glucose, TG, systolic blood pressure (SBP), and diastolic blood pressure (DBP), as well as with an improved lipid profile [222]. Zinc intake is associated with a significant decrease in body mass index, the apoA-I/apoB ratio, leptin, levels of oxidative stress and insulin resistance markers, as well as TG and LDL-C [223]. Both studies were conducted in individuals with diagnosed MetS (the zinc study in prepubertal children) using supplements; therefore, they do not allow conclusions to be drawn regarding the preventive efficacy of fortified foods. However, data on the effect of individual micronutrients on cardiometabolic risk factors remain contradictory, necessitating further randomized controlled trials [224,225].

Thus, fortification of mass-market food products with vitamins and minerals may be regarded as a direction for reformulation; however, its effects on MetS components require verification in human studies using reformulated products.

### 5.5. Polyphenols and Carotenoids

As noted in previous sections, oxidative stress and chronic low-grade inflammation are universal pathophysiological mechanisms underlying the initiation and progression of a wide range of metabolic disorders [226]. Fortification and targeted reformulation of food products with natural antioxidants, particularly polyphenols and carotenoids, are regarded as a promising approach capable of influencing oxidative stress, inflammation, and certain cardiometabolic markers. Such fortification qualifies as a biotechnological approach only when the respective compounds are produced or modified using biological systems, for example, through microbial synthesis of carotenoids, enzymatic biotransformation of polyphenols, or fermentation of plant raw materials; the addition of extracts or their encapsulation does not constitute biotechnology. However, the clinical significance of these effects varies depending on the compound type, dose, food matrix, bioavailability, and the level of evidence for a particular intervention.

Traditional plant extracts, spices, and culinary herbs have long been used in food products to enhance organoleptic characteristics and extend shelf life. These extracts are characterized by a high content of compounds with pronounced antioxidant activity, which include polyphenols (phenolic acids, flavonoids, anthocyanins, lignans, and stilbenes), carotenoids (xanthophylls and carotenes), as well as vitamins E and C [227]. According to predominantly preclinical and short-term clinical studies, these natural antioxidants may contribute to lowering blood pressure, improving the lipid profile, reducing glucose concentrations, and decreasing body weight, effects that are being considered in relation to MetS prevention [228].

Carotenoids are a large class of natural antioxidants found in many vegetables, foods, and other natural sources. Carotenoids have been characterized as having a broad spectrum of biological activity, including a potential protective effect (according to predominantly observational and experimental studies) against diabetes mellitus and cancer, as well as neurodegenerative, metabolic, and cardiovascular diseases [229,230,231,232]. Results from in vitro and preclinical studies indicate beneficial effects of carotenoid consumption on obesity and associated pathophysiological disorders, including metabolic inflammation, insulin resistance, and liver steatosis [233,234]. According to meta-analyses, intake of carotenoids, particularly β-carotene, α-carotene, lutein, and zeaxanthin, has been associated with improved glycemic parameters, especially in individuals with type 2 diabetes mellitus, as well as with reductions in body mass index (BMI), waist circumference, and total cholesterol levels and an increase in HDL-C levels [235].

Polyphenols are a group of bioactive compounds found in plant-based foods [236]. According to recent studies, a number of polyphenol-rich foods and beverages may exert beneficial effects on MetS manifestations [237]. The main sources of these compounds include green tea, red wine, grape seeds, berries, and dark chocolate, which contain both monomeric forms of flavanols—catechins—and their oligomers—proanthocyanidins [238].

#### Human Studies

A limited number of clinical studies have examined the effects of polyphenol-rich food consumption on anthropometric parameters such as body weight, body mass index, waist circumference, and fat mass in individuals with MetS. Overall, the results suggest potential beneficial effects of green tea, berries, and cocoa products, including reduced body mass index, fat mass, improved lipid profiles, and improved insulin resistance [239]. For example, regular green tea consumption three times daily for eight weeks was associated with improvements in several cardiometabolic markers in women with MetS [240]. In another study, daily consumption of 150 g of blueberries for six months was associated with favorable changes in several vascular and lipid parameters, including endothelial function, systemic arterial stiffness, HDL-C concentrations, and parameters reflecting nitric oxide (NO) bioactivity [241]. Furthermore, in a study in healthy volunteers, consumption of dark chocolate high in cocoa polyphenols immediately before an oral glucose tolerance test was associated with increased early insulin and GLP-1 secretion [242]. Because the studies of polyphenol-rich foods, green tea, and blueberries [239,240,241] were conducted in individuals with MetS, they characterize a therapeutic context; the preventive relevance of these effects in individuals without MetS has not been established. These results suggest the potential for polyphenol-containing berries and cocoa products to modulate individual components of MetS, but require further confirmation in independent long-term randomized trials.

The presented data allow polyphenols and carotenoids to be considered promising bioactive components capable of influencing individual components of MetS pathogenesis, primarily through modulation of oxidative stress, inflammatory processes, and lipid and carbohydrate metabolism. However, the level of evidence varies significantly depending on the class of compounds, food matrix, and study design. Despite the availability of results from randomized clinical trials and meta-analyses, a significant portion of the data remains based on mechanistic, preclinical, or short-term studies. Further research in this area should focus on elucidating the molecular mechanisms mediating the synergistic interactions of polyphenols and carotenoids within whole food matrices. This is essential for the evidence-based development of food fortification strategies aimed at reducing cardiometabolic risk factors at the population level.

## 6. Integration of Biotechnological Strategies into Dietary Patterns for the Prevention of Metabolic Syndrome

From the perspective of non-communicable disease prevention, a healthy diet is defined less by the properties of individual foods than by the context of their consumption, that is, their frequency, quantity, and place in the diet. Accordingly, key strategies for improving population health include ensuring dietary diversity with portion-size control and systematic food reformulation, which can alter the intake of nutrients of public health concern without requiring radical changes in established eating behavior [243].

**Figure 3:**
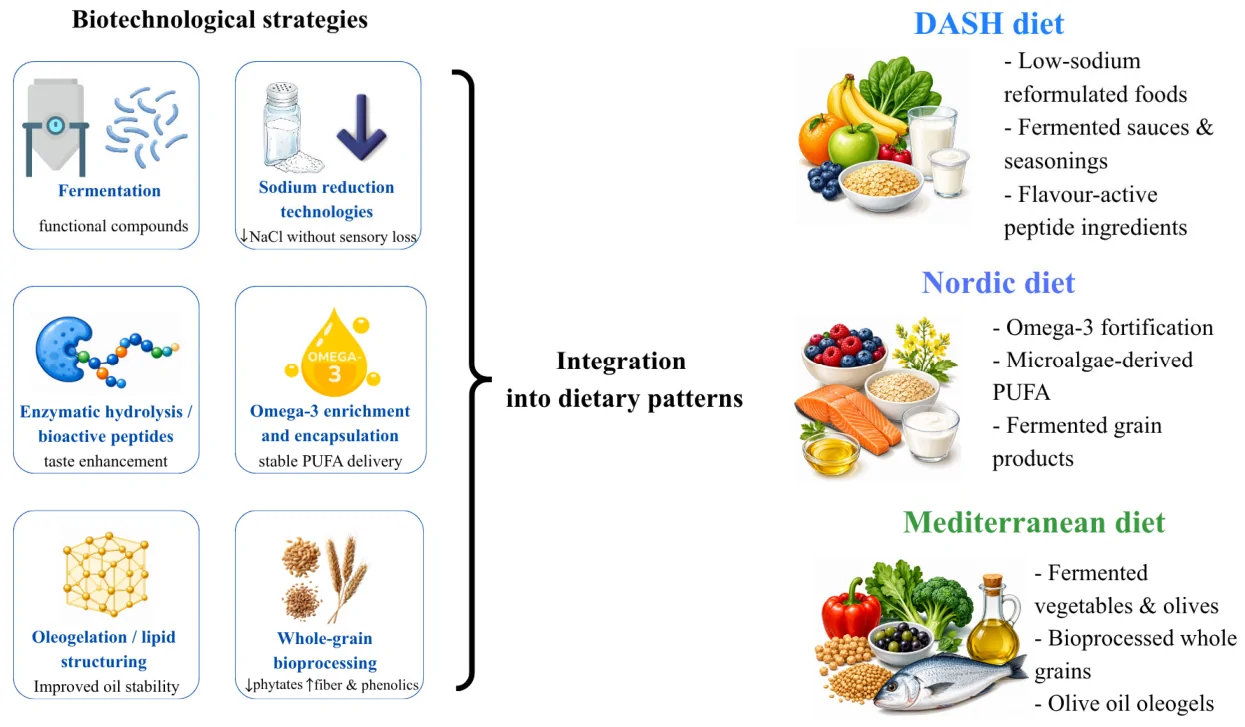
Conceptual framework illustrating potential points of integration of biotechnology-reformulated foods into established dietary patterns. The scheme illustrates potential opportunities for incorporating reformulated foods into different dietary patterns based on their modified nutrient profiles and their potential contribution to a more favorable dietary composition. The depicted relationships are hypothesis-generating and are intended to provide a conceptual framework for further research; they do not represent specific combinations of reformulated foods and dietary patterns that have been directly evaluated in intervention studies. The scheme should not be interpreted as evidence of the effectiveness of any particular combination of reformulated foods and dietary patterns for the prevention of MetS. ↓ - decrease; ↑ - increase.

Reformulation policies may contribute to transforming the food environment and creating conditions that support healthier food choices [244]. Because changes in the formulation of mass-market foods depend less on changes in individual dietary habits, reformulation is considered a potentially more acceptable tool for implementation within habitual diets [245]. This approach is consistent with the current understanding that overall dietary patterns, rather than only the ratio of individual macronutrients, play an important role in the prevention of chronic non-communicable diseases [246]. On this basis, it is of interest to examine conceptually how the biotechnological strategies reviewed could potentially be integrated into dietary patterns that have been shown in randomized trials and meta-analyses to have favorable effects on cardiometabolic risk factors. A conceptual scheme of such integration is presented in Figure 3. It illustrates hypothesized points of application for biotechnological strategies rather than findings from studies in which the corresponding combinations were directly evaluated. Accordingly, the integration strategies discussed below should be regarded as hypotheses to be tested rather than as evidence-based recommendations.

The Mediterranean diet (MedDiet) is characterized by a high intake of fruits, vegetables, nuts, legumes, cereals, fish, and other seafood. It has been consistently associated with reduced cardiovascular morbidity and mortality, as well as with a lower prevalence of MetS [247]. Biotechnological approaches could be used to improve the nutrient profile of individual foods within this dietary pattern. Many MedDiet ingredients are traditionally fermented, which may improve the functional properties of certain food components. However, the effect of fermented foods on the risk of metabolic disorders depends on product composition, dietary pattern, intake level, and study type [248]. Lactic acid fermentation of traditional MedDiet plant foods, such as artichokes, capers, and table olives, can modify their biochemical profile and, according to technological and in vitro studies, may increase the content and bioaccessibility of certain polyphenols [249]. Biotechnological processing of whole grains may improve protein and carbohydrate digestibility, reduce phytate content, increase the bioaccessibility of phenolic compounds, and increase soluble dietary fiber content [250]. The hypothesis that incorporating such products enhances the cardiometabolic effects of the MedDiet itself remains untested.

The Dietary Approaches to Stop Hypertension (DASH) diet, originally developed to lower blood pressure, may be considered a potential platform for integrating biotechnological sodium reduction strategies. In technological and sensory studies, fermented sauces and seasonings produced with *Zygosaccharomyces rouxii*, *Torulopsis halophilus*, and *Candida versatilis*, as well as plant protein-derived peptide hydrolysates that enhance salty taste perception, allowed the amount of added sodium chloride to be reduced without substantial deterioration of sensory properties [251,252]. It can be hypothesized that incorporating such products into the DASH diet may facilitate adherence to its sodium targets while maintaining the palatability of the diet. However, the effect of such integration on blood pressure has not been evaluated in intervention studies.

The Nordic diet is based on the consumption of whole grains, fruits, vegetables, legumes, nuts, fatty fish, lean meat, and low-fat dairy products, as well as on limited salt intake [253]. Potential directions for the biotechnological optimization of this dietary pattern include the use of cultivated microalgal oils as a source of ω-3 PUFAs for fortifying dairy and bakery products, as well as targeted fermentation of cereals to increase micronutrient bioavailability [254,255,256]. These approaches may improve the nutrient profile of the corresponding products; however, their impact on the metabolic effects of the Nordic diet has not been studied.

Thus, biotechnological reformulation can be considered a tool for improving the nutrient profile of foods within established dietary patterns rather than a stand-alone intervention. The hypothesis that incorporating such products into the MedDiet, DASH, or Nordic diet enhances the cardiometabolic benefits of these dietary patterns or facilitates adherence to them requires testing in intervention studies.

## 7. Safety, Regulatory, and Consumer-Acceptance Considerations

The practical application of biotechnological reformulation requires consideration not only of technological feasibility and metabolic rationale but also of the safety, tolerability, regulatory status, and consumer acceptance of reformulated products. These aspects are particularly important because such products are intended for regular consumption by broad population groups, including individuals with obesity, arterial hypertension, and impaired carbohydrate metabolism.

The regulatory status of the same ingredient differs substantially across jurisdictions and must be assessed separately for each jurisdiction, specification, and intended use. In the European Union, ingredients that were not consumed to a significant degree before 15 May 1997 are classified as novel foods; their market authorization is granted through inclusion in the Union list of novel foods (Commission Implementing Regulation (EU) 2017/2470) by decision of the European Commission, based on a scientific safety assessment conducted by the European Food Safety Authority (EFSA) [257]. Food additives are authorized under Regulation (EC) No 1333/2008, and health claims under Regulation (EC) No 1924/2006. In the United States, ingredients are used either as food additives authorized by U.S. Food and Drug Administration (FDA) regulations (Title 21 of the Code of Federal Regulations, CFR) or on the basis of conclusions that they are generally recognized as safe (GRAS). GRAS notification to the FDA is voluntary, and an FDA “no questions” response letter merely indicates that the agency does not object to the notifier’s conclusion; it is not equivalent to FDA approval.

The most pronounced differences between jurisdictions are observed for rare sugars. In 2025, the EFSA Panel on Nutrition, Novel Foods and Food Allergens (NDA) concluded that the safety of D-allulose as a novel food could not be established on the basis of the data provided [65]; at the time of manuscript preparation, D-allulose has not been included in the Union list, and other applications for its authorization are under evaluation. In the United States, D-allulose is used on the basis of several GRAS notices to which the FDA raised no questions (e.g., GRN 000400, 2012; GRN 000693, 2017; GRN 001188, 2024); according to the 2020 FDA guidance, it may be excluded from the total and added sugars declarations on the label, and its energy value may be taken as 0.4 kcal/g. D-Tagatose, by contrast, has been authorized in the EU as a novel food since 2005 and is used in the United States on the basis of GRAS notices (e.g., GRN 000977, 2021). Fructooligosaccharides are used in the United States on the basis of GRAS notices (e.g., GRN 000044); in accordance with the FDA’s 2018–2019 position, inulin-type fructans, galactooligosaccharides, type 2 resistant starch, resistant maltodextrin/dextrin, and cross-linked phosphorylated type 4 resistant starch may be declared as dietary fiber on the label. In the EU, chemically modified starches, including distarch phosphate and phosphated distarch phosphate, are classified as food additives (E1412 and E1413, respectively).

Ingredients used for sodium reduction have an established regulatory status: potassium chloride is authorized in the EU as food additive E508 and has affirmed GRAS status in the United States (21 CFR 184.1622), whereas 5′-ribonucleotides (IMP and GMP) are authorized in the EU as food additives E631 and E627, respectively, and in the United States as food additives (21 CFR 172.535 and 172.530). Oil from the microalga *Schizochytrium* sp., a source of DHA, is included in the Union list of novel foods and is used in the United States on the basis of GRAS notices (e.g., GRN 000137). For the MLM-type structured lipids discussed in this review, no specific EU authorizations or U.S. GRAS notices were identified; in the absence of a history of significant consumption before 15 May 1997, they are subject to assessment as novel foods. Bioactive peptides may be used as food ingredients; however, no health claims regarding their antihypertensive effects have been authorized in the EU, as EFSA did not establish a cause-and-effect relationship between the consumption of the tripeptides IPP and VPP and the maintenance of normal blood pressure [258]. For probiotic microorganisms, EFSA assigns qualified presumption of safety (QPS) status at the taxonomic unit level, whereas in the United States the status is determined for individual strains; no health claims for probiotics have been authorized in the EU. For ingredients produced using microbial production strains, the safety assessment additionally includes characterization of the production strain and confirmation of the absence of its viable cells in the final product.

Among auxiliary technologies, oleogelation using ethylcellulose relies on an authorized food additive (E462 in the EU; 21 CFR 172.868 in the United States), whereas engineered nanomaterials are classified as novel foods in the EU and require a separate safety assessment, and in the United States they are evaluated on a case-by-case basis. Thus, the regulatory status of an ingredient is not a fixed attribute: it depends on the jurisdiction, specification, manufacturer, and conditions of use and should be verified as of the date of development of a specific product.

Consumer acceptance determines the real-world impact of reformulation. Reducing the sugar, sodium, or saturated fat content improves the nutrient profile of a product; however, the practical value of reformulation depends on whether consumers regularly choose and consume such products. The acceptable degree of reformulation depends on the food matrix. According to a meta-analysis, salt reductions of approximately 37–40% in bread and 60–67% in processed meat products were not accompanied by a statistically significant decrease in overall acceptability, whereas for cheeses, a decrease in acceptability was observed at lower reduction levels [259]. Therefore, reformulation studies should report sensory analysis and acceptability assessed under repeated consumption alongside metabolic outcomes. In the absence of such data, conclusions regarding the scalability of reformulated products should be drawn with caution.

Thus, safety, regulatory status, and consumer acceptance are not secondary characteristics but necessary conditions for the effectiveness of reformulation at the population level. They define the limits of acceptable modification of the food matrix, the likelihood of regular product consumption, and the potential for scaling up reformulation. Accordingly, the evaluation of biotechnologically reformulated products should include not only metabolic markers but also measures of safety, tolerability, and sensory acceptability.

## 8. Limitations and Future Challenges

The technologies reviewed differ substantially in terms of human evidence, achievable intake levels, safety, sensory properties, storage stability, and cost, all of which determine their feasibility for practical implementation.

Among sugar reduction strategies, resistant starch and FOSs have been studied in both acute and long-term human trials (the latter in individuals with MetS or prediabetes), and their effects on metabolic markers were observed at intake levels achievable with bakery and cereal products. Both ingredients are heat-stable or moderately stable, have little effect on sensory properties at moderate doses, and are relatively inexpensive. However, FOSs undergo hydrolysis at low pH and high temperature and may cause gastrointestinal discomfort at high intakes. D-Allulose has been studied mainly in acute postprandial trials, and long-term data are lacking. Its intake is limited by gastrointestinal tolerance. It provides approximately 70% of the sweetness of sucrose, enhances Maillard browning during heating and storage, and remains more expensive than sucrose.

Among sodium reduction strategies, partial replacement of NaCl with KCl has the strongest evidence base, including long-term studies with hard cardiovascular endpoints. It is effective at 25–30% replacement, stable, and inexpensive. Its use is limited by bitter and metallic off-tastes at higher replacement levels and by the risk of hyperkalemia in individuals with chronic kidney disease or those taking potassium-sparing medications. In contrast, human data on reduced-sodium fermented sauces and pastes are limited to observational studies. Their absolute sodium content remains high, salt reduction may compromise their microbiological stability, and the formation of biogenic amines and ethyl carbamate requires monitoring. Their advantage is a pronounced umami taste.

For lipid modification strategies, no human studies were identified for the structured lipids reviewed, and data on oleogels are limited to a few acute postprandial studies. Enzymatic interesterification involves high production costs, and base oils rich in PUFAs are prone to oxidation during storage. Oleogels are prone to oil separation and polymorphic transitions and may alter texture and mouthfeel, although most structuring agents are approved food additives.

Bioactive peptides obtained by enzymatic hydrolysis or fermentation have been evaluated mainly in short-term human studies with inconsistent results, and no health claim regarding their antihypertensive effect has been authorized in the EU. Effective doses may require high product intake, and peptides may be degraded during digestion. Their use is further limited by bitterness, potential allergenicity, and susceptibility to Maillard reactions during storage.

Micro- and nanoencapsulation improve the stability of labile compounds and mask bitterness and astringency; however, human data are largely limited to foods fortified with vitamin D. The doses of encapsulated polyphenols used in animal studies are difficult to achieve through diet; nanoscale carriers require specific safety assessment; and high production costs and scale-up challenges limit commercial application. Biofortification is inexpensive and generally does not alter sensory properties; however, human data are limited to a few small studies, the content of the target nutrient depends on its accumulation in the plant and losses during cooking, and excessive intake (e.g., of iodine) requires monitoring.

Overall, KCl-based salt substitutes, resistant starch, and FOSs are currently the most suitable for large-scale implementation, whereas structured lipids, modified proteins, and nanoencapsulated bioactives require further clinical, toxicological, and economic evaluation. Notably, most of the studies reviewed did not report sensory evaluation, storage stability, or production cost data for the reformulated products, and data on the long-term safety of novel ingredients are lacking.

## 9. Conclusions

In conclusion, biotechnological food reformulation shows technological and biological potential for modifying cardiometabolic risk factors associated with MetS; however, the current evidence base remains limited and heterogeneous, which precludes extrapolating the findings for most of the strategies reviewed to population-level MetS prevention. Enzymatic processes, microbial bioconversion, and fermentation make it possible to modify the nutrient profile of widely consumed foods while potentially preserving acceptable sensory characteristics. However, data on the metabolic effects of such products have been obtained mainly in vitro and in preclinical models, which primarily support their biological plausibility. The available human studies are few, mostly short-term, and have been conducted either in healthy individuals with assessment of postprandial metabolic responses or in individuals with diagnosed metabolic disorders. Consequently, the available data provide only limited insight into the potential preventive value of reformulation. Moreover, the findings are heterogeneous and are largely based on surrogate metabolic markers.

In terms of the level of available evidence, approaches aimed at reducing sodium and free sugar content while maintaining product acceptability are the best supported, as reducing the intake of these nutrients is regarded as a component of population-based strategies for improving diet quality. In contrast, strategies that rely on additional physiological effects of biotechnologically introduced or generated bioactive components require independent verification in human studies. Building an evidence base for MetS prevention requires long-term randomized trials comparing reformulated foods with their conventional counterparts in individuals without diagnosed metabolic diseases, with assessment of clinically relevant endpoints. Until such data become available, biotechnological reformulation should be regarded as a promising tool for improving nutrient composition and the food environment, rather than as a proven stand-alone strategy for MetS prevention.

## Figures and Tables

**Table 2:** Biotechnological and auxiliary strategies for nutrient reformulation targeting MetS components.

Target Nutrient	Reformulation Strategy	Experimental System and Study Design	Food Matrix	Target Component of MetS	Metabolic Effects	References
In vitro studies						
Protein reformulation	Protein engineering with targeted release of bioactive peptides	Cell-free in vitro study: enzyme-inhibition and antioxidant assays	Modified concanavalin (CNV44)	Arterial hypertension, hyperglycemia, dyslipidemia, oxidative stress	Inhibition of ACE (IC_50_ 4.5 µg/mL), DPP-IV (IC_50_ 0.033 µg/mL) and HMGR; antioxidant and chelating effects	[151]
Animal studies						
Reduction in free sugar content	Synthesis of rare sugars (D-allulose/D-psicose)	Animal study: OLETF rats (spontaneous T2DM model) vs. LETO controls; 5% D-psicose in drinking water from 6 to 60 weeks of age	Drinking water supplemented with D-psicose (5% solution)	Insulin resistance, visceral obesity, dyslipidemia	Stabilization of blood glucose levels, reduced body weight gain in rats, attenuation of postprandial hyperglycemia and reduction in HbA1c	[152]
Reduction in sodium content	Microbial biotransformation of taste-active precursors	Animal study: estrogen-deficient (ovariectomized) rats	Lyophilized traditional soybean sauce (Kanjang) with a high content of *Bacillus* spp.	Obesity, insulin resistance, dyslipidemia	Reduced body weight gain and visceral fat accumulation; decreased fasting glucose and insulin levels; increased HDL-C and reduced LDL-C; modulation of the gut microbiota and increased butyrate production	[153]
Reduction in sodium content	Microbial biotransformation of taste-active precursors	Animal study: Sprague–Dawley rats with hypertension induced by a high-fat, high-salt diet	Chinese traditional fermented sauce (CTFSS)	Obesity, arterial hypertension, dyslipidemia	Reduction in body weight, epididymal fat and SBP; decreased serum levels of TC, TG, ALT, AST, renin, Ang II, ACE and aldosterone; improved Na^+^/K^+^ balance	[154]
Modification of the fatty acid profile	Enzymatic interesterification (synthesis of MLM-type structured lipids)	Animal study: Swiss mice with high-fat diet-induced obesity	Structured lipids derived from grape seed oil with incorporation of capric acid (C10:0)	Obesity, hyperglycemia, dyslipidemia, systemic inflammation	Reduced body weight gain and visceral adipose tissue mass; decreased plasma glucose and total cholesterol levels; improved glucose tolerance; reduced production of IL-6 and IL-10 by splenocytes	[155]
Modification of the fatty acid profile	Enzymatic interesterification and microencapsulation of MLM-type structured lipids	Animal study: mice fed a high-fat diet	Microencapsulated structured lipids (grape seed oil enriched with capric acid)	Obesity, hyperglycemia, dyslipidemia, hepatic steatosis	Reduced body weight gain and organ weights (liver, heart and testes); decreased serum insulin and leptin levels; improved glucose tolerance; reduced total and macrovesicular hepatocellular steatosis; decreased ALT, ALP, TG, and cholesterol levels	[156]
Protein reformulation	Microbial fermentation of proteins	Animal studies: rats with poloxamer 407-induced dyslipidemia, fructose-fed rats, spontaneously hypertensive rats	Fermented whey product MPM	Dyslipidemia, hyperglycemia, arterial hypertension	Reduced TG, total cholesterol, and LDL-C levels; increased HDL-C; improvement in the fasting glucose-to-insulin ratio; reduction in SBP	[157]
Protein reformulation	Enzymatic protein hydrolysis	Animal study: ApoE−/− mice, 12 weeks	Anchovy (*Engraulis encrasicolus*) collagen hydrolysate	Obesity, dyslipidemia, hepatic steatosis	Reduced body weight gain; twofold reduction in serum TC and reduction in TG levels; tenfold reduction in ALT and sixfold reduction in AST; twofold reduction in hepatic steatosis; 2.5-fold reduction in hepatic macrophage infiltration	[158]
Enhancement of polyphenol bioavailability	Microencapsulation (auxiliary technology)	Animal study: female Wistar rats, bilateral ovariectomy plus high-carbohydrate, high-fat diet (menopausal MetS model), 8 weeks	Microencapsulated mulberry fruit extract	Oxidative stress, insulin resistance	Reduced oxidative stress in the hippocampus and decreased acetylcholinesterase activity	[159]
Enhancement of polyphenol bioavailability	Microencapsulation (auxiliary technology)	Animal study: Wistar rats with MetS induced by 30% sucrose	Microencapsulated pomegranate juice	Arterial hypertension, hyperglycemia, dyslipidemia	Reduction in SBP and DBP; decreased blood glucose levels; reduced TC, LDL-C, and TG levels	[160]
Human studies (interventional and observational)						
Reduction in free sugar content	Synthesis of rare sugars (D-allulose)	Human intervention study: randomized, single-blind, crossover trial; healthy young women; acute (postprandial)	Chocolate with sucrose replaced by D-allulose	Hyperglycemia, dyslipidemia, obesity	Increased postprandial free fatty acid levels and reduced blood glucose and insulin levels	[161]
Reduction in free sugar content	Controlled synthesis of functional oligosaccharides (FOSs)	Human intervention study: randomized, double-blind, crossover trial; acute (postprandial)	Yacon syrup (14 g FOS)	Hyperglycemia, insulin resistance	Reduction in postprandial glycemia and insulin levels, most pronounced 30 min after intake; positive association between the insulin response and the relative abundance of *Actinobacteria* and *Bifidobacteriales*	[162]
Reduction in free sugar content	Incorporation of resistant carbohydrate forms	Human intervention study: randomized, double-blind, crossover trial; acute (postprandial)	Baked breakfast bar with corn starch replaced by tapioca-based resistant starch (RS4)	Hyperglycemia, insulin resistance	Reduction in postprandial glycemia (iAUC0–120 by 22%, *p* < 0.05) and postprandial insulinemia (iAUC0–120 by 37%, *p* < 0.05)	[163]
Reduction in sodium content	Partial replacement of NaCl with KCl (75:25) (non-biotechnological approach, for comparison)	Human intervention study: stepped-wedge cluster-randomized trial; community setting	Salt substitute mixture (75% NaCl + 25% KCl)	Arterial hypertension	Reduction in SBP by 1.29 mmHg and DBP by 0.76 mmHg; 51% reduction in the risk of arterial hypertension among normotensive participants	[164]
Reduction in sodium content	Microbial biotransformation of taste-active precursors	Observational human study: cross-sectional analysis of a large hospital-based cohort	Fermented soybean paste (Jang)	Obesity, arterial hypertension, dyslipidemia, hyperglycemia	Inverse association between high Jang intake (≥4.9 g/day) and the risk of MetS: reduced waist circumference, decreased fat mass, lower serum glucose levels, and lower prevalence of hypo-HDL cholesterolemia	[165]
Modification of the fatty acid profile	Structuring of oleogels from plant oils (coconut and sunflower oils) (auxiliary technology)	Human intervention study: randomized, controlled, crossover trial; healthy men; acute (postprandial)	Rice porridge supplemented with oleogel	Hyperglycemia, dyslipidemia	Improvement in postprandial glycemia and carbohydrate and fat oxidation; attenuation of the hypertriglyceridemia typically associated with liquid oils	[166]
Protein reformulation	Enzymatic protein hydrolysis	Human intervention study: randomized, double-blind, placebo-controlled, crossover trial; adults with MetS (therapeutic context)	Egg white hydrolysate	Arterial hypertension	Reduction in fasting pulse pressure and DBP	[167]
Enhancement of iodine and molybdenum content	Biofortification	Human intervention study: randomized, single-blind, controlled trial	Lettuce	Dyslipidemia	Increased HDL-C, reduced TG levels, decreased AST and ALT levels, and increased PYY	[168]
Enhancement of vitamin D bioavailability	Nanoencapsulation and fortification (auxiliary technology)	Human intervention study: quadruple-blind, randomized, placebo-controlled trial; adults with MetS (therapeutic context)	Low-fat yogurt	Oxidative stress, systemic inflammation	Increased serum vitamin D levels; reduced high-sensitivity C-reactive protein and pro-oxidant–antioxidant balance	[169]

Evidence derived from in vitro and animal studies should be interpreted as mechanistic or preclinical and should not be directly extrapolated to clinical efficacy in humans without confirmation in randomized controlled trials. Non-biotechnological approaches (encapsulation, oleogelation, and partial replacement of NaCl with KCl) are labeled separately in the table and are included as auxiliary technologies or for comparison; they are not considered biotechnological strategies. Human intervention study—interventional study in human participants (randomized, crossover, or acute postprandial); observational human study—cohort, case–control, or cross-sectional study; animal study—experiment in laboratory animals; cell-free in vitro study—chemical, enzymatic, or simulated-digestion assays without living cells; cell-based in vitro study—experiments in cultured cells. MPM—malleable protein matrix; T2DM—type 2 diabetes mellitus; OLETF—Otsuka Long-Evans Tokushima Fatty; LETO—Long-Evans Tokushima Otsuka; IL—interleukin; RS4—type 4 resistant starch; HbA1c—hemoglobin A1c; iAUC—Incremental Area Under the Curve; SBP—systolic blood pressure; DBP—diastolic blood pressure; TC—total cholesterol; TG—triglyceride; ALT—alanine aminotransferase; AST—aspartate aminotransferase; Ang II—angiotensin II; ACE—angiotensin-converting enzyme; ALP—alkaline phosphatase; DPP-IV—dipeptidyl peptidase IV; HMGR—3-hydroxy-3-methylglutaryl coenzyme A reductase; PYY—peptide YY.

## Data Availability

No new data were created or analyzed in this study. Data sharing is not applicable to this article.

## Abbreviations

The following abbreviations are used in this manuscript:

MetS	Metabolic syndrome
GLP-1	Glucagon-like peptide-1
GIP	Glucose-dependent insulinotropic polypeptide
mTORC1	Mammalian target of rapamycin complex 1
AMPK	AMP-activated protein kinase
GCN2	General control nonderepressible 2
SCFAs	Short-chain fatty acids
PYY	Peptide YY
NAFLD	Non-alcoholic fatty liver disease
FOS	Fructooligosaccharide
GOS	Galactooligosaccharide
GMP	Guanosine 5′-monophosphate
IMP	Inosine 5′-monophosphate
sdLDL	Small dense low-density lipoprotein
LDL-C	Low-density lipoprotein cholesterol
HDL-C	High-density lipoprotein cholesterol
TG	Triglyceride
VLDL	Very-low-density lipoprotein
SFA	Saturated fatty acid
TFA	Trans-fatty acid
apoB-100	Apolipoprotein B-100
apoA-I	Apolipoprotein A-I
MCFAs	Medium-chain fatty acids
LCFAs	Long-chain fatty acids
MLM	Medium-long-medium structured lipid
PUFA	Polyunsaturated fatty acid
DHA	Docosahexaenoic acid
EPA	Eicosapentaenoic acid
HOMA-IR	Homeostatic model assessment of insulin resistance
QUICKI	Quantitative insulin sensitivity check index
HbA1c	Hemoglobin A1c
DASH diet	Dietary Approaches to Stop Hypertension diet
MedDiet	Mediterranean diet
EFSA	European Food Safety Authority
FDA	Food and Drug Administration
GRAS	Generally Recognized as Safe
